# Simulations of working memory spiking networks driven by short-term plasticity

**DOI:** 10.3389/fnint.2022.972055

**Published:** 2022-10-03

**Authors:** Gianmarco Tiddia, Bruno Golosio, Viviana Fanti, Pier Stanislao Paolucci

**Affiliations:** ^1^Department of Physics, University of Cagliari, Monserrato, Italy; ^2^Istituto Nazionale di Fisica Nucleare (INFN), Sezione di Cagliari, Monserrato, Italy; ^3^Istituto Nazionale di Fisica Nucleare (INFN), Sezione di Roma, Rome, Italy

**Keywords:** computational neuroscience, spiking neural networks, NEST simulation, working memory, short-term plasticity (STP)

## Abstract

Working Memory (WM) is a cognitive mechanism that enables temporary holding and manipulation of information in the human brain. This mechanism is mainly characterized by a neuronal activity during which neuron populations are able to maintain an enhanced spiking activity after being triggered by a short external cue. In this study, we implement, using the NEST simulator, a spiking neural network model in which the WM activity is sustained by a mechanism of short-term synaptic facilitation related to presynaptic calcium kinetics. The model, which is characterized by leaky integrate-and-fire neurons with exponential postsynaptic currents, is able to autonomously show an activity regime in which the memory information can be stored in a synaptic form as a result of synaptic facilitation, with spiking activity functional to facilitation maintenance. The network is able to simultaneously keep multiple memories by showing an alternated synchronous activity which preserves the synaptic facilitation within the neuron populations holding memory information. The results shown in this study confirm that a WM mechanism can be sustained by synaptic facilitation.

## 1. Introduction

Working memory (WM) is a cognitive process that is able to hold and manipulate information for a short time. It is involved in a vast number of cognitive tasks (Miller et al., 1960; Baddeley and Hitch, 1974; Cowan, 1998; Golosio et al., 2015) which span from speech to visual and spatial processing. However, the WM capacity, i.e., the ability to hold multiple memories at the same time, is limited to a few items depending on the type of information (Miller, 1956; Cowan, 2001, 2010). Different from long-term memory, WM is a transient phenomenon, and it is also believed that it does not entail structural changes to the network.

A classic procedure for studying WM relies on the so-called delay response tasks. In such a framework, a stimulus is presented for a short time and the related execution of the task can take place only after a delay period. During the delay period, it is experimentally observed, especially in the prefrontal cortex (PFC), a neuronal selective spiking activity able to maintain the information previously presented by the stimulus (Funahashi et al., 1989; Goldman-Rakic, 1995; D'Esposito and Postle, 2015). When this activity is somehow suspended (e.g., because of a noise stimulus during the delay period or a too long delay), the task is not correctly executed.

The first computational models assumed that this peculiar activity could be entirely maintained with prior long-term synaptic modifications so that when a stimulus was given to the network, the population encoding for the presented stimulus exhibited persistent spiking activity (Hebb, 1949; Hopfield, 1982; Brunel, 2000). Thus, according to these models, the information was only stored in the spiking activity. However, experimental evidence shows that memory can be also maintained when the enhanced activity is interrupted, suggesting that information is not only stored in the population's spiking activity (Stokes, 2015) but also that WM processes can exhibit discrete periodic bursts instead of a persistent activity (Honkanen et al., 2014; Lundqvist et al., 2016).

In this framework, many studies were conducted to enlighten the role of synaptic plasticity in WM (Barak and Tsodyks, 2014), and some of the proposed models rely on short-term synaptic plasticity, especially on short-term facilitation (Barak and Tsodyks, 2007; Mongillo et al., 2008; Hansel and Mato, 2013; Rolls et al., 2013). Indeed, it has been observed that the PFC shows marked short-term facilitation (Wang et al., 2006), suggesting that this form of plasticity can have a significant link with WM tasks. The work of Rolls et al. (2013) shows that employing synaptic facilitation enables a spiking network to maintain a relevant number of memories at the same time, whereas the same network lacking this kind of plasticity can maintain far fewer memories. Moreover, in Hansel and Mato (2013), it is described that the non-linearity of short-term facilitation is essential for displaying a reasonable persistent activity able to retain memory during a delay period. One of the models that posit a dominant role for synaptic facilitation in WM is Mongillo et al. (2008) model, which shows that a spiking network with synaptic facilitation is able to exhibit a bi-stable regime in which it can autonomously retain memories with periodic spiking activity without a significant firing rate increase. Thus, according to this model, memories are stored in a synaptic fashion, with spiking activity functional for synaptic facilitation upkeep. The model is further developed by Mi et al. (2017) to study how WM capacity can be modulated by short-term synaptic plasticity and the network's external excitation.

In addition, Mongillo et al. (2008) presented a simple mean-field model describing the firing rate behavior of an excitatory population modulated by short-term plasticity. This model has also been explored in Cortes et al. (2013), in which the short-term synaptic plasticity can lead to irregular and chaotic dynamics, facilitating transitions between network states and thus being one possible mechanism responsible for complex dynamics in cortical activity. Furthermore, Taher et al. (2020) developed a neural mass model with short-term synaptic plasticity based upon the dynamics of a network of quadratic integrate and fire (QIF) neurons. Interestingly, this study was able to qualitatively reproduce the results of Mongillo et al. (2008) using facilitated synapses. Also, the maintenance of multiple memories was explored and presented an analytic expression for the WM capacity based on the work of Mi et al. (2017), in agreement with the value observed in the simulations of the network of QIF neurons.

Recently, Fiebig and Lansner (2016) (refer to also Fiebig et al., 2020) proposed a spiking network model based on a fast expression of Hebbian plasticity, in which memory is retained by oscillatory bursts. The authors of the above-mentioned study proposed a synaptic plasticity model based on a Hebbian learning rule supplemented by a short-term plasticity mechanism. This kind of implementation can enable a network to learn new memory representations, whereas using non-Hebbian plasticity needs prior long-term network training.

In this work, we implement the spiking network model described in Mongillo et al. (2008) using the NEST simulator. We show that the network exhibits totally comparable results with respect to the original article, underlining the role of short-term synaptic plasticity in WM tasks. The memory specific response of the network can be regulated by modulating the spontaneous activity. Moreover, the network is capable of maintaining multiple items at the same time and the number of items that can be maintained can be tuned by changing the short-term plasticity parameters.

## 2. Materials and methods

### 2.1. Short-term synaptic plasticity

In this section, short-term plasticity and its phenomenological description are introduced. For further details, please refer to Markram et al. (1998), Tsodyks et al. (1998, 2000), Barak and Tsodyks (2007), and Barri and Mongillo (2022).

Short-term plasticity (STP) is a mechanism in which the synaptic efficacy temporarily changes with a timescale on the order of hundreds or thousands of milliseconds. This phenomenon is regulated by the amount of synaptic resources (i.e., the neurotransmitters) available in the synapse at the moment of spike emission and by the calcium levels in the presynaptic terminal.

Indeed, the amount of neurotransmitters a synapse can contain is limited, and the emission of a spike diminishes the number of neurotransmitters available in the presynaptic terminal for further stimulation. Without synaptic activity, the amount of available neurotransmitters in the presynaptic terminal returns to its baseline level. This mechanism is called short-term depression (STD). Moreover, the spike arrival at the presynaptic terminal elicits an influx of calcium ions that is responsible for the release of the vesicles in which neurotransmitters are stored. Higher calcium concentration in the terminal leads to a higher fraction of neurotransmitters released. This mechanism is called short-term facilitation (STF). The neurotransmitter release is then followed by a mechanism of calcium removal from the presynaptic terminal to restore its baseline concentration.

The coupling of these two phenomena leads to a temporary modulation of the synaptic efficacy (i.e., short-term plasticity), which can show STD-dominated or STF-dominated behaviors. The former can be observed when the mechanism of neurotransmitter restoration is slower with respect to the mechanism of residual calcium removal after spike emission and vice versa. To give a phenomenological description of STP, we can define τ_*d*_ as the time constant of the process of neurotransmitter restoration and τ_*f*_ the time constant for the calcium removal mechanism. Thus, we can observe STD-dominated dynamics when τ_*d*_>τ_*f*_ and STF-dominated dynamics when τ_*d*_ < τ_*f*_.

The synaptic efficacy modulation led by STP can be described by the following phenomenological model: let *x* be the normalized amount of available resources into the presynaptic terminal and let *u* be the fraction of resources used in a spike emission. The spike arrival to the synaptic terminal rises the variable *u* by a quantity *U*(1−*u*) (so that *u* remains normalized), and the amount of resources released is equal to *ux*. Considering a synapse connecting the presynaptic neuron *i* and the postsynaptic neuron *j*, this dynamics can be described by the following equations (Mongillo et al., 2008):


(1) $$\begin{array}{l} \frac{du_{i,j}}{dt}=-\frac{u_{i,j}-U}{\tau_{f}}+U\left(1-u_{i,j}\right)\underset{s}{\sum }\delta \left(t-t_{s}^{\left(i\right)}\right)\\ \frac{dx_{i,j}}{dt}=\frac{1-x_{i,j}}{\tau_{d}}-u_{i,j}x_{i,j}\underset{s}{\sum }\delta \left(t-t_{s}^{\left(i\right)}\right) \end{array}$$

where δ(·) is the Dirac delta function and the sum is over the spike times $t_{s}^{\left(i\right)}$ of the presynaptic neuron *i*. The synaptic modulation takes place during the spike emission, so that


(2) $$\begin{array}{l} J_{i,j}\left(t\right)=J_{i,j}^{\left(abs\right)}u_{i,j}\left(t-{\hat{\delta }}_{i,j}\right)x_{i,j}\left(t-{\hat{\delta }}_{i,j}\right) \end{array}$$

where $J_{i,j}^{\left(abs\right)}$ is the absolute synaptic efficacy for the synapse connecting neurons *i* to neuron *j* and ${\hat{\delta }}_{i,j}$ is the synaptic delay. Thus, when a spike is fired, the synaptic efficacy is described by the product *Jux*.

### 2.2. Spiking network model

This section describes the spiking network model implemented in this work, following the Supplementary material of Mongillo et al. (2008).

The network is composed of *N*_*E*_ excitatory and *N*_*I*_ inhibitory leaky integrate-and fire (LIF) neurons with exponential postsynaptic currents. The sub-threshold dynamics of the LIF neuron model is described by the differential equation


(3) $$\begin{array}{l} \tau_{m}\frac{dV_{j}}{dt}=-V_{j}+R_{m}\left(I_{j}^{exc}+I_{j}^{inh}+I_{ext,j}\right) \end{array}$$

where τ_*m*_ is the membrane time constant, *V*_*j*_ is the neuron's membrane potential, *R*_*m*_ is the membrane resistance, $I_{j}^{exc}$ and $I_{j}^{inh}$ represent the excitatory and inhibitory synaptic currents received as input from the connections within the other neurons of the network and *I*_*ext, j*_ represents the external input to the network.

The network external input is modeled with Gaussian white noise currents defined by the following


(4) $$\begin{array}{l} I_{ext,j}\left(t-{\hat{\delta }}_{j}\right)=\mu_{ext}+\sigma_{ext}G_{k}\;\text{for}\;k\Delta t_{ng}\leq \left(t-{\hat{\delta }}_{j}\right)\leq \left(k+1\right)\Delta t_{ng} \end{array}$$

In particular, the noise is approximated by a piecewise constant current with mean μ_*ext*_ and standard deviation σ_*ext*_, with constant current during time intervals of length Δ*t*_*ng*_ = 1 ms. Denoting the index of the time interval with *k*, for each interval, the current is given by μ_*ext*_+σ_*ext*_*G*_*k*_, with *G*_*k*_ a random number extracted from a standard Gaussian distribution. The term ${\hat{\delta }}_{j}$ indicates the delays.

The synaptic current shown in Equation (3) is the sum of the contributions given by the connections with the neurons of the network, and it is characterized by excitatory and inhibitory contributions defined as $I_{j}^{exc}\left(t\right)$ and $I_{j}^{inh}\left(t\right)$, respectively. Thus, the synaptic input for a neuron *j* of the network, with exponential postsynaptic currents, is given by the following equations for excitatory and inhibitory currents, respectively:


(5) $$\begin{array}{l} \tau_{exc}\frac{dI_{j}^{exc}}{dt}=-I_{j}^{exc}+\underset{i}{\sum }\alpha J_{i,j}\left(t\right)\underset{s}{\sum }\delta \left(t-t_{s}^{\left(i\right)}-{\hat{\delta }}_{i,j}\right)\\ \tau_{inh}\frac{dI_{j}^{inh}}{dt}=-I_{j}^{inh}+\underset{i}{\sum }\alpha J_{i,j}\underset{s}{\sum }\delta \left(t-t_{s}^{\left(i\right)}-{\hat{\delta }}_{i,j}\right) \end{array}$$

where *i* is the index of the presynaptic neurons targeting the neuron *j*. τ_*exc*_ and τ_*inh*_ represent the time constant of the excitatory and inhibitory synaptic currents, respectively. In this model, τ_*exc*_ = τ_*inh*_ = 2 ms. ${\hat{\delta }}_{i,j}$ is the synaptic delay for the synapse connecting neurons *i* and *j*. All the delays are uniformly distributed between 0.1 and 1.0 ms. The time dependence of the synaptic efficacy *J*_*i, j*_ is only due to short-term plasticity modulation, and it is described in Equation (2), whereas synapses not modulated by the STP dynamics have fixed values of *J*_*i, j*_. Since in this model, only the connections between excitatory neurons employ short-term plasticity, the connections with inhibitory neurons do not show a time dependent synaptic efficacy. In addition, since the synaptic efficacies $J_{i,j}^{\left(abs\right)}$ are expressed in mV, a factor α is needed in order to be consistent with the units of Equation (5). This term derives the variation of current input needed to elicit a unit of variation of the postsynaptic potential (refer to Supplementary material for its derivation).

The excitatory neurons are organized into five selective populations, each of which includes a fixed fraction of neurons, and a non-selective population that includes the rest of the excitatory neurons of the network. In the base model, the selective populations of excitatory neurons have no overlap, so a neuron cannot belong to different selective populations. However, in an extension of the model, it is possible to simulate it with overlapping selective populations. In such a framework, the neurons belonging to each selective population are randomly chosen from the whole excitatory population, enabling the possibility of having neurons belonging to more than one selective population.

**Figure 1:**
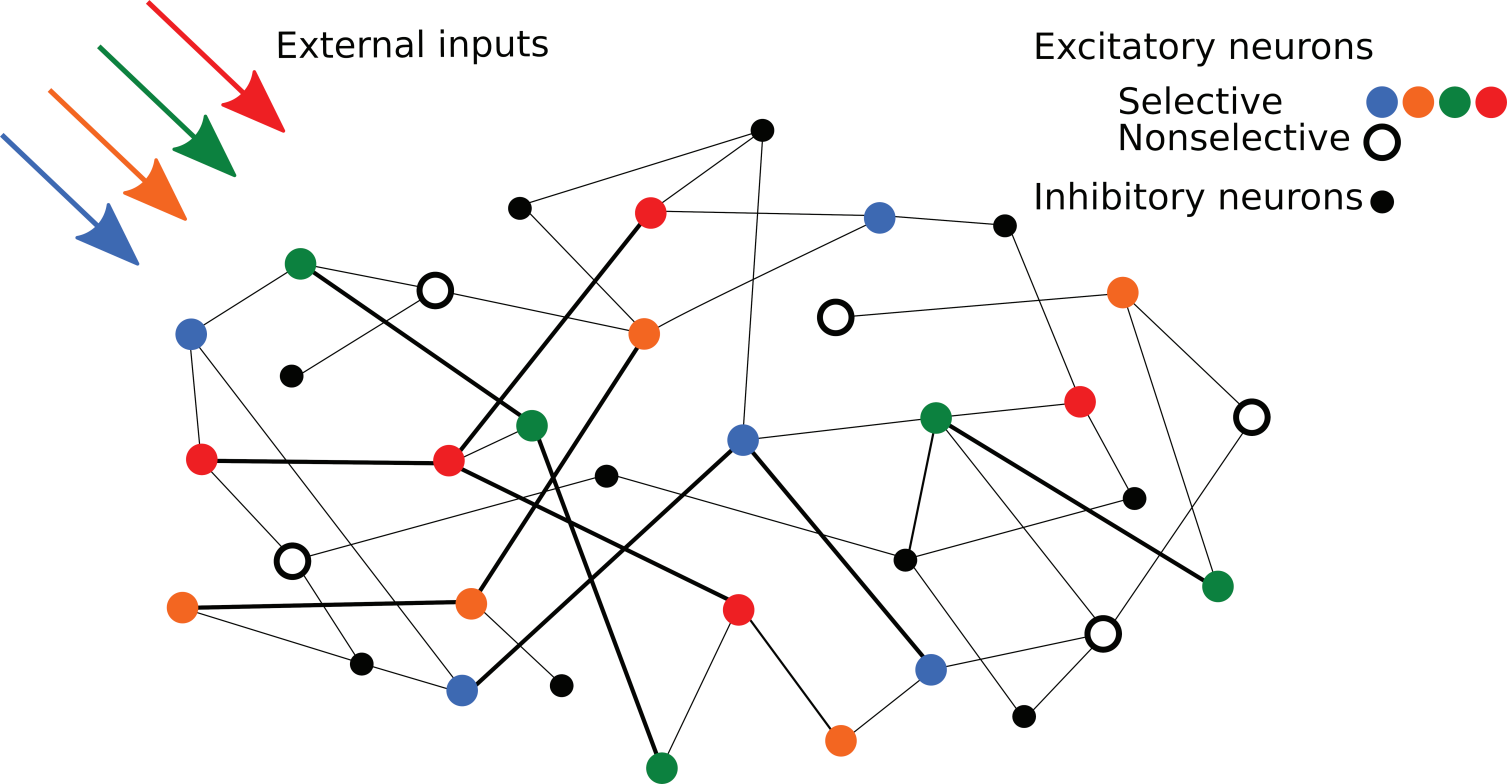
Schematic representation of the network. Colored circles represent excitatory neurons of different selective populations, whereas the black open circles represent excitatory neurons of the non-selective population. Black circles represent inhibitory neurons. Strengthened connections are represented by thicker black lines.

Regarding network's connectivity, short-term plasticity is implemented in all the excitatory-to-excitatory connections using the same time constants in order to show synaptic facilitation. These connections are thus characterized by the STP variables *x* and *u* and a weight *J*, which represents the absolute synaptic efficacy. The weights of the connections within excitatory neurons belonging to the same selective population assume a potentiated value *J*_*p*_, emulating the result of prior long-term Hebbian learning. On the other hand, connections between excitatory neurons belonging to different selective populations, or linking a selective population with the non-selective one, are set to a baseline value *J*_*b*_. The rest of the excitatory-to-excitatory connections have the baseline synaptic efficacy except for the 10% of them that show the potentiated value. While the excitatory-to-excitatory connections show STP dynamics, the other connections are static connections with hard-coded synaptic weights. The overall connectivity is structured so that each neuron of the network receives a fixed amount of connections from the network's populations, both excitatory and inhibitory, with non-specific inhibitory connectivity. The possibility of having more than one synapse with the same two neurons is also enabled. A simplified scheme of the spiking network architecture is depicted in Figure 1.

Such a network is able to store memories by exploiting the STP mechanism of the excitatory-to-excitatory connections. In fact, when a signal targets a selective population increasing its spiking activity, the synapses connecting neurons of the targeted population remain facilitated for a time in the order of τ_*f*_. The connections between neurons inside the pre-stimulated selective population are potentiated because *J*^(*abs*)^ is relatively large due to prior long-term Hebbian learning (having $J^{\left(abs\right)}=J_{p}$) and also because of the STP modulation driven by *u* and *x*. In particular, the variable *u* shows a slow decay to its baseline value, whereas *x* grows rapidly toward its asymptotic value. On the other hand, connections between neurons belonging to other selective populations are relatively weaker because they lack short-term potentiation, while the connections between neurons belonging to different selective populations are weaker because they have not been previously potentiated by long-term Hebbian learning (with $J^{\left(abs\right)}=J_{b}$ in this case). A similar effect could be driven by a random fluctuation of neuron activity, inducing an autonomous winner-take-all (WTA) mechanism, according to which the selective excitatory population with the highest firing rate stimulates the inhibitory population eliciting a suppression of the spiking activity of the other excitatory populations (Coultrip et al., 1992) due to the global inhibition. Indeed, the global inhibition is granted by the non-specific inhibitory connectivity, in agreement with experimental observations (Fino and Yuste, 2011). This mechanism decreases the amount of available resources *x* and increases the value of *u* across the pre-stimulated selective population. When *x* returns close to its baseline value, since the value of *u* is still relatively high, the connection strength becomes large enough to trigger again the WTA mechanism. This process can be reactivated periodically, and the period of reactivation is related to the dynamics of *x* and in particular to the time constant of the synaptic depression τ_*d*_.

All the parameters used for the spiking network simulations, together with an in-depth description of the network connectivity, are reported in the Supplementary material.

## 3. Results

In this section, we present the results of the spiking network simulations performed using the NEST simulator (version 3.1) (Deepu et al., 2021).

The network is composed of 8,000 excitatory and 2,000 inhibitory LIF neurons with exponential postsynaptic currents, whose dynamics are described by Equations 3, 4, and 5 [refer to also Equations 1, 2, 4, and 5 in Burkitt (2006) and Equation 3 in Hanuschkin et al. (2010)]. The neuron model differs from the one employed in the original work, as in Mongillo et al. (2008) a LIF neuron model with the instantaneous rise and decay times for postsynaptic currents is employed. As discussed in Section 2.2, the excitatory population is further divided into five selective populations of 800 neurons each and a non-selective population that includes the rest of the excitatory neurons. All excitatory-to-excitatory connections follow an STP dynamics whereas the rest of the connections have fixed synaptic efficacies.

The simulations are performed using a time step of 0.05 ms, with the system of Equations (3) and (5) integrated following the exact integration scheme of Rotter and Diesmann (1999) and assuming that the external current *I*_*ext, j*_ is a piecewise constant over time intervals of width Δ*t*_*ng*_. This is an additional difference with respect to Mongillo et al. (2008), in which both Equation (3) describing the neuron sub-threshold dynamics and Equation (1) describing the STP mechanism are integrated using the Euler scheme. In the NEST implementation presented here, Equation (1) is not integrated at every time step, but the values of the variables *x*_*i, j*_ and *u*_*i, j*_ are analytically obtained whenever a spike is emitted by the presynaptic neuron *i*. In particular, having two consecutive spikes emitted at times *t*_*s*_ and *t*_*s*+1_ and knowing *x*(*t*_*s*_) and *u*(*t*_*s*_), the evolution of variables is computed as follows:


(6) $$\begin{array}{l} x\left(t_{s+1}^{-}\right)=1+\left(x\left(t_{s}^{+}\right)-1\right)e^{-\left(t_{s+1}-t_{s}\right)/\tau_{d}}\\ u\left(t_{s+1}^{-}\right)=U+\left(u\left(t_{s}^{+}\right)-U\right)e^{-\left(t_{s+1}-t_{s}\right)/\tau_{f}}\\ u\left(t_{s+1}^{+}\right)=u\left(t_{s+1}^{-}\right)+U\left(1-u\left(t_{s+1}^{-}\right)\right)\\ x\left(t_{s+1}^{+}\right)=x\left(t_{s+1}^{-}\right)-u\left(t_{s+1}^{+}\right)x\left(t_{s+1}^{-}\right) \end{array}$$

where *t*_*s*_ represents the spike time, while $t_{s}^{-}$ and $t_{s}^{+}$ represent the times immediately before and immediately after the spike emission, respectively. More formally, $x\left(t_{s}^{-}\right)$ and $u\left(t_{s}^{-}\right)$ can be intended as the left-side limits:


(7) $$\begin{array}{l} x\left(t_{s}^{-}\right)=\underset{\epsilon \to 0}{\lim}x\left(t_{s}-\epsilon \right)\;\text{with}\epsilon \in {\mathbb{R}}^{+}\\ u\left(t_{s}^{-}\right)=\underset{\epsilon \to 0}{\lim}u\left(t_{s}-\epsilon \right)\;\text{with}\epsilon \in {\mathbb{R}}^{+} \end{array}$$

while $x\left(t_{s}^{+}\right)$ and $u\left(t_{s}^{+}\right)$ can be intended as the right-side limits:


(8) $$\begin{array}{l} x\left(t_{s}^{+}\right)=\underset{\epsilon \to 0}{\lim}x\left(t_{s}+\epsilon \right)\;\text{with}\epsilon \in {\mathbb{R}}^{+}\\ u\left(t_{s}^{+}\right)=\underset{\epsilon \to 0}{\lim}u\left(t_{s}+\epsilon \right)\;\text{with}\epsilon \in {\mathbb{R}}^{+} \end{array}$$

Because of the discontinuity due to the spike emission, in general, the left-side and right-side limits differ from each other for the variables *x* and *u*. On the other hand, the exponential functions appearing in the first two lines of Equation (6) are continuous everywhere; therefore, the left and right limits are equal to each other for these functions. Therefore, the modulation led by short-term plasticity shown in Equation (2) is given by $u\left(t_{s+1}^{+}\right)x\left(t_{s+1}^{-}\right)$, thus considering variable *x* immediately before the spike emission and the variable *u* updated at the time of the spike emission as described in Tsodyks et al. (1998). Only after spike emission, the variable *x* is decreased because of neurotransmitter release. This order of update stems from the fact that the presynaptic spike triggers facilitation (i.e., the increase of the variable *u*) just before the spike emission to the postsynaptic neuron. Equation (6) is implemented in the NEST simulator with the tsodyks3_synapse model, a modified version of the NEST synapse model tsodyks2_synapse model, which describes the STP dynamics according to Equation (1) as well but modulates the synaptic efficacy using the term $u\left(t_{s+1}^{-}\right)x\left(t_{s+1}^{-}\right)$. Indeed, such a difference in the implementation can be relevant, especially with neurons having low firing rates (Gast et al., 2021), with tsodyks3_synapse model showing higher modulated synaptic efficacies than tsodyks2_synapse model (refer to Supplementary material for a comparison between the two synaptic models). In this model, the STP timescales are set so that the network shows synaptic facilitation, in fact, τ_*d*_ = 200 ms and τ_*f*_ = 1, 500 ms in agreement with the parameters chosen in Mongillo et al. (2008).

All the simulations begin with a time period of 3,000 ms in which only the background input is injected into the whole network in order to allow the network to enter its baseline state illustrating spontaneous activity. This stimulation, as well as all the other external signals, is created using the NEST noise_generator, which injects a Gaussian white noise current as described in Equation (4). The background input targets both excitatory and inhibitory neurons with different mean current values. Later in this section, it will be shown how network behavior can be modulated by changing excitatory background activity.

After the network reaches its spontaneous activity, an additional current, designed as a Gaussian white noise current which sums up to the background input, is injected only into a selective population for 350 ms. As a result, an item is loaded into the model. This signal, called item loading, increases the synaptic activity of the target population and thus permits a temporary strengthening of synaptic efficacies by changing the STP variables *u* and *x* across the connections of neurons belonging to the target population. Thus, even after the end of the item loading signal, the loaded memory can be maintained especially because of the slow decaying dynamics of the variable *u* due to synaptic facilitation.

**Figure 2:**
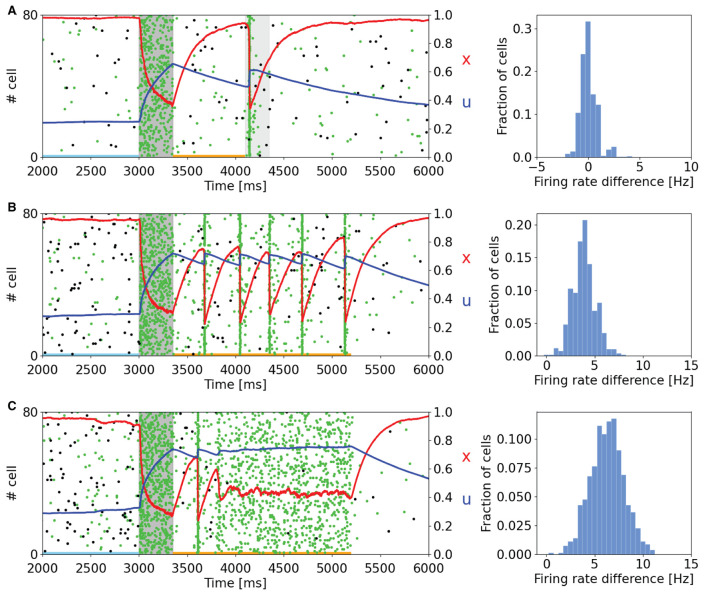
Raster plots of a subset of neurons of a targeted selective population (green) and a non-targeted one (black) for different values of background input. Averaged STP variables (*x* in red and *u* in blue) of the synaptic connections belonging to the target population are also shown. (Left) **(A)** The network, after the end of the injection of the item loading signal (gray shading) does not show a significant difference in spiking activity until the injection of a nonspecific input targeting the whole excitatory population (lighter gray shading). **(B)** Network behavior with increased excitatory background input. In this case, the network autonomously reactivates the memory by showing periodic synchronized events. The network returns to its spontaneous state by diminishing the background input. **(C)** Network behavior with further increased background input. Here, the network shows an asynchronous enhanced spiking activity of the loaded memory. As in **(B)**, the network returns to its spontaneous state when background input diminishes. (Right) Histograms represent the difference in firing rate between the delay period and the spontaneous state for the selective population targeted by the item loading signal. The rate at the spontaneous state is calculated before the injection of the item loading signal (sky blue line at the bottom of the panels). In **(A)**, the delay period is intended as the time between the end of the item loading stimulus and the beginning of the nonspecific external input. In **(B,C)**, the delay period is defined as the time between the end of the item loading signal and the decrease in the external background input (here shown at 5.2 s for both the panels). The delay period is indicated with an orange line at the bottom of the left panels.

As can be seen in Figure 2, the memory specific response of the network depends on the background activity level of the excitatory neurons. This figure shows the raster plot of two selective populations, one targeted by the additional current which loads the item and a non-targeted one, together with the STP variables *x* and *u* averaged over the connections outbound from the neurons of the targeted selective population. In Figure 2A, to reactivate a memory, a supplemental external signal targeting the entire excitatory population is given. Although this external signal is nonspecific, only the population in which the memory was previously restored responds with the emission of a single synchronized activity, called a population spike. The network can also autonomously exhibit a memory specific spiking activity when a higher excitatory background current is injected (Figures 2B,C). In Figure 2B, the selective population which receives the item loading stimulation shows an autonomous and synchronous emission of population spikes. It should be noted that after each population spike, the STP variable *u* increases and returns to similar values reached at the end of the item loading signal injection, interrupting the exponential decrease due to the calcium removal mechanism and thus enabling a new population spike to emerge. This behavior, together with the fast exponential growth of available resources described by the variable *x*, leads to a new stable state for the network together with the one representing spontaneous activity. To interrupt the network persistent activity, we set the excitatory background current to the value of Figure 2A. In Figure 2C the background input is further increased, and the network spontaneously shows an asynchronous higher rate activity. In this state, the memory is maintained in both spiking and synaptic form since the STP parameters reach stable values during the high activity state followed by a population spike. As in the previously described state, the network could pass from the memory specific activity state to the spontaneous state by diminishing the background input. Indeed, without the diminishing of the background input, the network would continue to behave showing the asynchronous higher rate activity or the synchronous emission of population spikes. The values of the background current used in Figure 2 are reported in the Supplementary Table S2. Moreover, we quantitatively estimated the difference in firing rate for the targeted selective population between the delay period and the spontaneous activity state. The difference in firing rate for a neuron population is obtained by measuring the spike-count rate for each neuron of the population at two-time intervals. Naming *r*_*s*_ the firing rate measured during the spontaneous activity state and *r*_*d*_ the firing rate measured during the delay period, the firing rate difference for a neuron *i* of the population is


(9) $$\begin{array}{l} \Delta r^{\left(i\right)}=r_{d}^{\left(i\right)}-r_{s}^{\left(i\right)}=\frac{N_{d}^{\left(i\right)}}{\Delta t_{d}}-\frac{N_{s}^{\left(i\right)}}{\Delta t_{s}} \end{array}$$

where *N*^(*i*)^ is the number of spikes emitted by neuron *i* in a certain time interval Δ*t*. Those values are obtained for each neuron of the targeted selective population and are collected in the histograms on the right side of Figure 2. In Figure 2A, the delay period is defined as the time between the end of item loading and the beginning of the nonspecific signal, whereas in the other panels, it is identified between the end of the item loading and the decreasing of the external input (happening at 5.2 s for both panels). The time intervals related to the spontaneous activity and the delay period are indicated with horizontal lines (sky blue and orange, respectively) in the left panels of Figure 2. It is possible to notice that in Figure 2A there is no significant difference in firing rate, and a relevant part of the network shows a decrease in firing rate during the delay period. In Figures 2B,C, we observed an increase in firing rate of about 4 and 7 Hz, respectively, with an average baseline firing rate of about 0.7 Hz. These changes in firing rate are lower with respect to the ones shown in network models relying only on persistent activity to show WM behavior such as Brunel (2000) and they are in agreement with experimental measures on single-cell activity during the delay period (Shafi et al., 2007), according to which the changes in firing rate are mostly below 5 Hz.

In comparison with the work of Mongillo et al. (2008), the network simulated with NEST shows qualitatively similar results, with comparable behavior when modulating the background input targeting the excitatory neurons. However, we noticed some relevant differences with respect to the original work. For instance, on the left side of Figure 2B, it is possible to see that the time interval between adjacent population spikes is around 300 ms, whereas in Mongillo et al. (2008), this value is closer to 200 ms, same order of τ_*d*_. Furthermore, while the behavior of the variable *u* is mostly comparable to the one shown in the original article, the behavior of the variable *x* shows a considerably higher drop of the averaged variable in correspondence to a population spike. This pronounced drop in the value of *x* is probably the reason for the difference in the time interval between the two population spikes previously mentioned.

**Figure 3:**
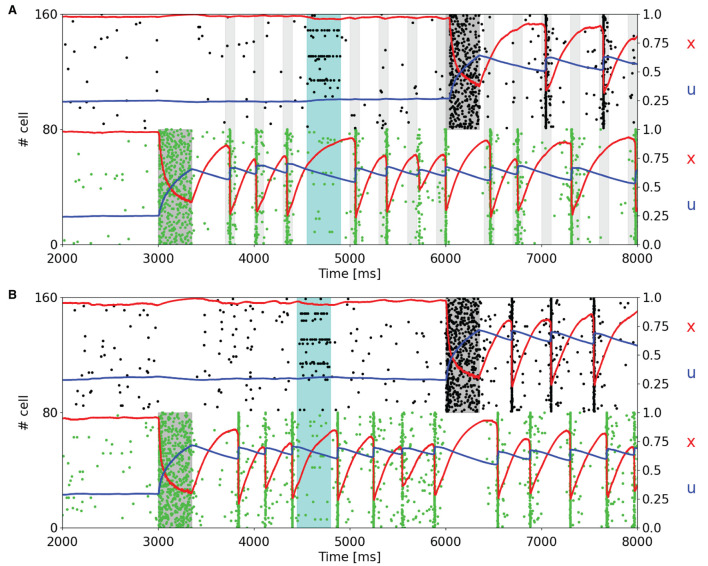
Raster plots of a subset of neurons of two targeted selective populations when two items are loaded into the network. An additional noise (cyan shading), which targets 15% of the excitatory neurons, is injected to test the network's robustness. **(A)** Network showing a single stable activity state injected with a periodic readout signal targeting all the excitatory neurons. After the second memory is loaded into the network, the populations show alternating population spikes. **(B)** Network in the bi-stable regime showing synchronous spiking activity. Here, the network does not receive the periodic input since the synchronous activity autonomously shows up after the item loading. After the second memory is loaded, the population spikes of the stimulated selective populations alternate, refreshing the synaptic variables in order to maintain the synchronous spiking activity.

Since one of the main features of a WM network is the holding of multiple information, we load two items into two different selective populations at different times to analyze the behavior of the STP variables of the targeted populations and the capacity of such a network of maintaining multiple items. Figure 3 shows a subset of two selective populations targeted by the item loading signal in the single stable state regime (Figure 3A) and in the regime showing synchronous and autonomous reactivation (Figure 3B), obtained using the same values of background input used in Figures 2A,B, respectively. Moreover, in both simulations noise is given to a fraction of all the excitatory neurons in order to check the robustness of the network state. The noise signal is designed as the item loading one but targets the 15% of the excitatory neurons randomly. In Figure 3A, the reactivation of the selective populations is enabled by a periodic nonspecific input (with a period of 300 ms). It can be noticed that in this framework, the two targeted selective populations do not emit the population spikes during the same periodic readout signal, but they alternate in order to reach suitable values of STP variables to enable the emission of a population spike in the following readout signal. This peculiar behavior can also be seen when the network autonomously shows synchronous spiking activity (Figure 3B). In this case, similarly to Figure 3A, the synchronous activity of the targeted selective populations is alternated, increasing the average value of *x* for a population when the other one is emitting the population spike. However, in Figure 3B, this mechanism is completely autonomous. In both the network states, the slow dynamics of *u* has a key role in holding the information, in particular when another selective population shows a higher spiking activity. In addition, it can be observed that the higher spiking activity of a selective population inhibits the other populations. This is due to the network's connectivity which enables a winner-take-all mechanism, i.e., the competition between different populations through a mechanism of global inhibition, as previously described. For this reason, it is not possible to correctly load multiple items at the same time, and it is not possible to have population spikes from different selective populations at the same time. As can be seen in Figure 3A, even if the readout signal targets all the selective populations, only the targeted selective population which has the highest STP-modulated synaptic efficacy is capable of emitting a population spike, inhibiting the excitatory neurons of the competing selecting populations.

The behavior of the network in Figure 3 is totally comparable with respect to the results shown in Mongillo et al. (2008). The main differences that emerge are related, as stated before, to the dynamics of the STP variable *x*, which shows a more pronounced drop when neurons show synchronous firing activity. We slightly increased the time interval between two consecutive stimulations in Figure 3A, from 250 to 300 ms, to make the STP variable *x* recover enough to enable the synchronous activity response as in Figure 2A. Shorter time intervals between subsequent readout signals could result in stimulation that leads to a population spike right after the end of the stimulus.

**Figure 4:**
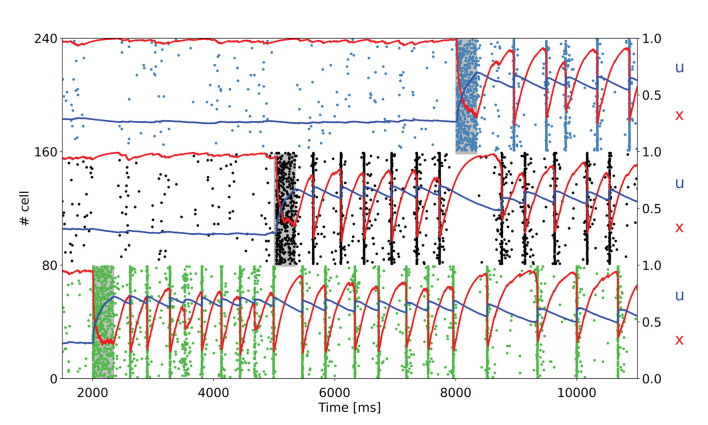
Raster plot of a subset of neurons of three targeted selective populations when three items are loaded into the network. The network is in the bi-stable regime showing synchronous spiking activity. Item stimuli are loaded into the network at 3, 000, 6, 000, and 9, 000 ms.

To further test the network capacity of maintaining multiple items at the same time, we perform simulations with an additional item loading signal targeting a third selective population for the network state showing synchronous spiking activity (same value of background input as in Figure 2B). The raster plot, together with the averaged STP variables for the three targeted selective populations, is depicted in Figure 4.

As shown in Figure 4, the network is able to maintain three selective populations in the persistent activity state similar to Figure 3B such that, when all the items are loaded, population spikes alternate within the population keeping appropriate values for the STP variables. Moreover, it should be noted that each selective population in the synchronous spiking activity regime diminishes the average value for the STP variable *u* when other items are loaded into the network. This behavior is clearly visible for the first selective population in Figure 4. Indeed, this is due to the increased distance between population spikes related to the activity of the other targeted selective populations. For an increasing number of items loaded, this can lead to a loss of synchronicity, since a persistent activity state needs to maintain a relatively high values of *u*. In this regard, we verified that an additional item loaded into the network causes the mentioned loss of synchronicity, thus such a network is able to maintain up to three items at the same time. However, an increase in the value of τ_*f*_ leads to a slower decay of the variable *u*, enabling the loading of more items into the network. For instance, with τ_*f*_ = 2, 000 ms, the network is able to maintain four items at the same time, and with τ_*f*_ = 3, 000 ms, all the five selective populations can alternate their population spikes (refer to Supplementary material). We also simulate a larger spiking network with ten selective populations in order to see whether a further increase in τ_*f*_ would enable the upkeep of more items. We show in the Supplementary material that an opportune choice of the synaptic parameters can enable the upkeep of even seven items simultaneously (i.e., the early estimation of the WM capacity proposed by Miller, 1956). Indeed, to simulate an analogous model with ten selective populations there is a need for a larger network having a similar ratio between selective, non selective, and inhibitory populations. For this purpose, we simulated a network with 20,000 LIF neurons, with ten selective populations each composed of a similar number of neurons to that of the network described above. The parameters used to perform these simulations are presented in the Supplementary materials.

**Figure 5:**
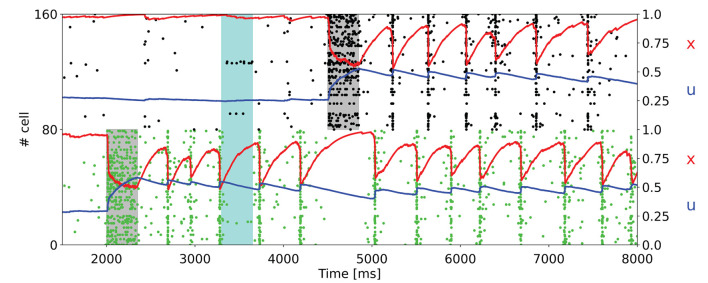
Raster plot for a simulation with overlapping populations. Here only a subset of the two targeted selective populations is shown. An additional noise (cyan shading) is also injected. The network is in the bi-stable regime showing synchronous spiking activity.

Hitherto, we presented the results of the simulations for the model with non overlapping populations, ergo an excitatory neuron can only belong to a selective population at most. To verify the network's behavior in more realistic conditions, we also performed simulations in which there is the possibility of having overlaps between the selective populations. Figure 5 shows the raster plot of a simulation with the same parameters used in Figure 3B, but with overlapping populations. Here, the population spikes are less synchronized, and not all the neurons belonging to the selective population emit a spike during the synchronous spiking activity. For this reason, the STP variable *x* drops caused by the population spikes are less pronounced. To obtain a qualitatively similar behavior with respect to the network with non overlapping populations, the value of the potentiated synaptic efficacy *J*_*p*_ has been slightly increased.

## 4. Discussion

In this work, we have reproduced a WM spiking network model proposed by Mongillo et al. (2008), in which the short-term synaptic plasticity has a key role in the network capability of memories upkeep. We have performed the simulations using the spiking network simulator NEST and following the network description and the parameters shown in the original work. We also modified the NEST synaptic model describing short-term plasticity to be consistent with Tsodyks et al. (1998).

Indeed, the spiking network model proposed here has some differences with respect to the original one of Mongillo et al. (2008). First, we employed a LIF neuron model with exponential postsynaptic currents, whereas the original one used a LIF model with the instantaneous rise and decay times for postsynaptic currents. Furthermore, the neuron model is integrated following the exact integration method of Rotter and Diesmann (1999), with synaptic variables for the neuron *i* synapses updated when the neuron *i* emits a spike, as mentioned in the Results section. The implementation of the STP dynamics follows Equations (1) and (6). In the original model, both neuron and synapse dynamics are integrated using the Euler scheme. The implementation of the STP dynamics further differs with respect to the original work. In fact, in Mongillo et al. (2008) and Mi et al. (2017), the absolute synaptic efficacy is modulated using the values of the variables *u* and *x* immediately before the emission of the spikes. As described in Equation (6), the implementation used in this work considers the value of the variable *x* immediately before the spike emission, but with the variable *u* updated at the time of the emission of the spike, in agreement with Tsodyks et al. (1998). This change in the implementation leads to higher modulated synaptic efficacies for the implementation employed (refer to also Gast et al., 2021 for a comparison of the two different implementations in a network of QIF neurons), and thus can be responsible for the more pronounced drop of the variable *x* noticed in the spiking model presented in this study. Despite these differences, we were able to obtain a similar behavior with respect to the original model by slightly adjusting some parameters. However, other parameters were missing, like the integration time step. We decided to set a time step of 0.05 ms, verifying that lower or higher time steps do not entail significant changes in the network behavior (refer to Supplementary material). Moreover, the connection scheme in the NEST simulator opens to the possibility of having multiple connections with the same two neurons or also self connections. In the simulations presented here, we enabled both multiple and self connections and we verified that the dynamics of the network do not change significantly when these options are disabled (refer to Supplementary material). Furthermore, we also performed simulations with the same neuron model integrated with a different integration scheme with respect to the exact integration method of Rotter and Diesmann (1999). Specifically, we employed the stochastic Runge-Kutta method, more suitable in the presence of noise signals modeled as the background input employed in this network. We found that the results of the simulations are totally comparable with respect to the one presented in the manuscript, as shown in the Supplementary material after a description of the integration method.

Figure 2, showing the raster plot of a selective population targeted by an item loading signal and a non-targeted population, exhibits totally comparable results with respect to the original work. The response of the network can be tuned by changing the excitatory background activity, and different behaviors can thus be shown: with relatively low background activity (refer to Figure 2A), the network needs an additional excitatory signal to exhibit a memory specific response, whereas an increase in the background input can lead to a synchronous (Figure 2B) or an asynchronous (Figure 2C) higher rate persistent activity. Such responses are driven by short-term plasticity, which temporarily modulates the synaptic efficacy within the connections belonging to neurons of the targeted selective population. In particular, the slow dynamics of calcium release from the synaptic terminal grant the temporary growth of the average synaptic efficacy of the population, leading to temporary storage of the memories in a synaptic fashion. Additionally, as it is possible to see in the histograms of Figure 2, the increase in firing rate for the targeted population is relatively modest. Besides, the model can stop showing the synchronous or the asynchronous higher rate activity only by diminishing the background input current, thus was able to maintain memories for extremely long periods of time, as shown in the Supplementary material. Indeed, bifurcation analysis of a single population rate model discussed in the Supplementary material of Mongillo et al. (2008) shows that for constant inputs, the network can show two possible behaviors: a steady state with a constant rate and a limit cycle solution corresponding to a periodic train of population spikes, which is consistent from what is observed in Figure 2B. A further study (Cortes et al., 2013) shows that, besides these stable solutions, the system can exhibit another class of states with highly irregular and chaotic-like dynamics, denoted as Shilnikov chaos.

Moreover, Figure 3 exhibits the ability of the network of maintaining multiple items at the same time and also the robustness of the network to external noise, here modeled as an item loading signal targeting a fraction of the whole excitatory neurons. It should be noted that the synchronous spiking activity of the first stimulated population, once an additional population is targeted by the item loading signal, interrupts and then alternates when the latter starts showing the synchronous persistent activity. This behavior enables the maintenance of two items at the same time. In addition, the current parameters enable the network to maintain up to three populations in the synchronous activity state (Figure 4). We verified that stimulating a fourth selective population makes the network lose synchronicity and the alternation of the so-called population spikes for the stimulated selective populations. However, we noticed that an increase in τ_*f*_ and, more generally, a change in the synaptic parameters enable the upkeep of a higher number of items at the same time (refer to Supplementary material). Indeed, as observed in Mi et al. (2017), in which a spiking network developed using the same framework as Mongillo et al. (2008) is simulated, the number of items that can be stored into the WM network (i.e., the WM capacity) can be modulated by a different choice of the synaptic parameters and of the background input. Additionally, the network has been simulated with partially overlapped selective populations to show the network's behavior in a more realistic condition. Figure 5 shows a totally comparable behavior with respect to an analog simulation with non overlapped populations, except for the fact that not every neuron of the selective population emits a spike during a population spike. Also, the population spike shows less synchronized spiking activity.

Regarding WM capacity, Mi et al. (2017) provides an analytical expression for estimating the maximum number of items that can be maintained in WM (see also Taher et al., 2020 for a similar derivation). This number is determined by the ratio between *T*_*max*_, i.e., the maximal period of the limit cycle of the network and *t*_*s*_, i.e., the time interval between two successive population spikes. Indeed, the maximal period of the limit cycle is only dependent on STP parameters and can be expressed by Mi et al. (2017)


(10) $$\begin{array}{l} T_{max}≃\tau_{d}\ln\;\frac{\tau_{f}/\tau_{d}}{1-U} \end{array}$$

Using the parameters employed to produce Figure 4, in which up to three items can be stored in WM, *T*_*max*_≃445 ms, whereas the time separation between the population spike, once the three items have been loaded into the network, is approximately *t*_*s*_≃160 ms, with the ratio between these times being


$$N_{c}\approx T_{max}/t_{s}≃2.8$$

not far from the number of items stored at the same time, confirming the generality of the analytical estimation proposed in Mi et al. (2017). We also performed a similar calculation for a network able to store up to seven memories in the Supplementary material, with comparable results.

Indeed, the model presented in this work is consistent with several experimental observations. For instance, in Wolff et al. (2015, 2017) showed that, during the delay period, the information held in memory can be reactivated by a non-specific stimulus (as in Figure 2A). This result is also shown by Rose et al. (2016), in which transcranial magnetic stimulation produced a brief reactivation of the held item. Moreover, the silent dynamics can lead to interference between information from different trials (Kilpatrick, 2018), and the relation between STP dynamics and the so-called serial effects in WM tasks has recently been explored in Kiyonaga et al. (2017) and Barbosa et al. (2020). Furthermore, as already mentioned in the Results section, the firing rate changes between the spontaneous state and the delay period shown in the right panels of Figure 2 are in agreement with single-cell firing rate, which is mostly below 5 Hz and only rarely can reach values greater than 10 Hz (Shafi et al., 2007). Indeed, since a higher spiking activity would be more metabolically demanding, this behavior makes the model energetically efficient highlighting the importance of activity-silent dynamics during WM tasks and also enables a multiple memory maintenance having populations emitting bursts at different times, in agreement with Lundqvist et al. (2016).

On the other hand, this model has some limitations. The main one being that it assumes a prior long-term Hebbian learning. The way items are encoded in selective populations is extremely simplified, as all the connections within the same population have equal synaptic strength. Furthermore, this value remains constant during the simulation. A more realistic model would be a combination of long-term and short-term plasticity, enabling the learning of new items.

Working memory is responsible for the brain's ability to temporarily maintain, manipulate, and integrate information from different sensory systems (auditory, visual, etc.) during the performance of a wide range of cognitive tasks, such as learning, reasoning, and language comprehension. Indeed, this mechanism is not only useful, but can have an essential role in robotics and autonomous systems in general. For instance, WM implementation could lead to autonomous systems with cognitive capabilities closer to the human ones, enabling the possibility of learning through interactions between humans, or learning from few examples integrating information from different sensory inputs in a similar way that humans do. Recent work has shown that the use of a WM component in robotic models can be useful to emulate many human-like cognitive functions, ranging from episodic memory, imagination and planning (Balkenius et al., 2018), language development (Giorgi et al., 2021b), and language grounding into actions and perceptions in embodied cognitive architectures (Giorgi et al., 2021a).

In conclusion, in this study, we reproduced a spiking network that shows typical WM behavior driven by short-term synaptic plasticity. This mechanism leads to a robust and energetically efficient behavior since the items loaded into the network can be maintained with a relatively low change in the population firing rate. The model, developed using the well-known spiking network simulator NEST, is available on an online repository (refer to Data Availability Statement). The NEST implementation of the model can pave the way to further studies aimed at a better understanding of WM mechanisms and of the link between short-term synaptic plasticity and long-term cognitive processes such as learning (Capone et al., 2019; Golosio et al., 2021).

## Data availability statement

The original contributions presented in this study are publicly available. The Python implementation of the spiking network model can be found at https://github.com/gmtiddia/working_memory_spiking_network. The NEST version of the model used to perform the simulations, provided with the synapse model used in this work, can be found at https://github.com/gmtiddia/nest-simulator-3.1.

## Author contributions

GT wrote the manuscript. GT, BG, and PP performed the simulations. GT, BG, VF, and PP revised the manuscript. BG and PP supervised the project. All authors have read and approved the final manuscript.

## Funding

This study was supported by the European Union's Horizon 2020 Framework Programme for Research and Innovation under Specific Grant Agreements No. 945539 (Human Brain Project SGA3), No. 785907 (Human Brain Project SGA2), and the INFN APE Parallel/Distributed computing Laboratory. We acknowledge the use of Fenix Infrastructure Resources, which are partially funded by the European Union's Horizon 2020 research and innovation programme through the ICEI project under grant agreement No. 800858.

## Conflict of interest

The authors declare that the research was conducted in the absence of any commercial or financial relationships that could be construed as a potential conflict of interest.

## Publisher's note

All claims expressed in this article are solely those of the authors and do not necessarily represent those of their affiliated organizations, or those of the publisher, the editors and the reviewers. Any product that may be evaluated in this article, or claim that may be made by its manufacturer, is not guaranteed or endorsed by the publisher.
