# Supplementary material for: Simulations of working memory spiking networks driven by short-term plasticity
Source: Front Integr Neurosci. 2022 Oct 3;16:972055. doi: 10.3389/fnint.2022.972055 (PMC9574057; doi:10.3389/fnint.2022.972055)
Supplement: Supplementary file 1 [file Data_Sheet_1.pdf]

Supplementary Material

1 SPIKING NETWORK PARAMETERS AND IN-DEPTH MODEL DESCRIPTION

Here we show the network parameters used to implement the spiking network. In Table S1 are shown the parameters needed to build the entire network, whereas Table S2 shows the parameters of the external stimuli, followed by an in-depth description for a better understanding of the network architecture.

**Neuron parameters**	**Excitatory**	**Inhibitory**
V_{th} - spike threshold	20.0 mV	20.0 mV
V_{reset} - reset potential	16.0 mV	13.0 mV
E_L - resting potential	0.0 mV	0.0 mV
V_m - membrane potential at t=0 ms	0.0 mV	0.0 mV
C_m - membrane capacity	250.0 pF	250.0 pF
τ_m - membrane time constant	15.0 ms	15.0 ms
τ_{syn} - synaptic current time constant	2.0 ms	2.0 ms
τ_{ref} - refractory period	2.0 ms	2.0 ms
**Network parameters**	**Values**	
f - fraction of neurons in a synaptic population	0.1	
p - number of selective populations	5	
c - connectivity level	0.2	
N - number of neurons	8000	2000
**Synaptic parameters**	**Values**	
J_{IE} - synaptic efficacy E $\rightarrow$ I	0.135 mV	
J_{EI} - synaptic efficacy I $\rightarrow$ E	0.25 mV	
J_{II} - synaptic efficacy I $\rightarrow$ I	0.2 mV	
J_b - baseline efficacy for E $\rightarrow$ E synapses	0.10 mV	
J_p - potentiated efficacy for E $\rightarrow$ E synapses	0.45 mV	
γ_0 - probability of having a potentiated efficacy	0.10	
$\hat{\delta}$ - synaptic delays	$0.1 - 1.0$ ms uniformly distributed	
**Short-term plasticity parameters**	**Values**	
U - baseline utilization factor	0.19	
u - probability of release at t=0 ms	0.19	
x - available resources at t=0 ms	1.0	
τ_F - recovery time of utilization factor	1500 ms	
τ_D - recovery time of synaptic resources	200 ms	

Table S1. Parameters used to build the spiking network. In case of overlapping populations, $J_p = 0.49$ mV.

As mentioned in the manuscript, the network is composed of N_E excitatory and N_I inhibitory neurons (here 8000 and 2000, respectively). The excitatory neurons are further divided in p selective populations containing fN_E neurons. Neurons are then connected following the `fixed_indegree` rule, thus all the neurons of the postsynaptic population have a fixed number of connections with random neurons from the presynaptic populations. Each neuron receives cfN_E connections from each of the p selective populations,

**Background excitatory current**	**E**	**I**
η_{ext} - mean membrane potential difference elicited by external current	variable	20.5 mV
Σ_{ext} - standard deviation of potential difference elicited by external current	1.0 mV	1.0 mV
Δt_{ng} - interval of current update	1.0 ms	

**Item loading signal parameters**	**Values**
T_{cue} - duration	350 ms
A_{cue} - contrast factor	1.15

**Nonspecific reactivating signal**	**Values**
T_{reac} - duration	250 ms
A_{reac} - contrast factor	1.05

**Periodic reactivating signal**	**Values**
T_{period_reac} - duration	100 ms
Period	300 ms
A_{period_reac} - contrast factor	1.075

Table S2. Parameters used to build the external stimuli. The values of mean external stimulus given to the network to show the behavior of Figure 2 are 22.7 mV, 23.7 mV and 24.1 mV for panel (A), (B) and (C) respectively. The other plots in the manuscript use the same values of external current employed for Figure 2 panels when reproducing a state activity represented in the respective panel. Those values, in potential units, are used to compute average μ_{ext} and standard deviation σ_{ext} of a current stimulus able to elicit a difference in the membrane potential with given mean and standard deviation. Contrast factors are defined so that the overall average external current injected into the network is equal to $A\mu_{ext}$ with a standard deviation of σ_{ext}.

then it receives $c(1-fp)N_E$ from the excitatory non-selective population and finally cN_I from the inhibitory population. Thus, each neuron of the network receives $c(N_E + N_I)$ connections from other neurons of the network. The synaptic delays were computed using the `nest.random.uniform` function to be uniformly distributed between 0.1 ms and 1.0 ms. When the connections are between excitatory neurons, if the neurons belong to the same synaptic population, the synapse assumes a potentiated efficacy J_p, otherwise assumes the baseline value J_b. Additionally, if the presynaptic neuron belong to the non-selective population, the synaptic efficacy assumes the potentiated valued with probability γ_0 (i.e. the 10% of them show potentiated efficacy, the rest have the baseline value J_b). The synaptic efficacy for the other connections in the model are shown in Table S1. Furthermore, the synaptic efficacy is expressed in mV, meaning that it is defined to generate a post-synaptic potential of amplitude J. To get the value in pA (i.e. the input needed in NEST synapse dictionary) needed to elicit a postsynaptic potential difference J, we used a specific factor α, which represents the current increase that elicits a unit of postsynaptic potential-variation for an integrate-and-fire neuron model with exponential post-synaptic currents. The derivation of the factor α, shown in Equation S17, is illustrated in a subsequent section of the Supplementary Material. Moreover, the network parameter `overlap` is a Boolean value which enables the possibility of having partially overlapped selective populations. In this case, the neurons belonging to each selective population are chosen randomly within the whole excitatory population, so that a neuron can thus belong to more than one selective population.

The external stimuli, as described in Section 2.2 of the manuscript, are designed as Gaussian white noise currents of mean μ_{ext} and standard deviation σ_{ext}. The current values change every Δt_{ng}. The parameters η_{ext} and Σ_{ext} presented in Table S2 are used to compute the values of μ_{ext} and σ_{ext} needed to elicit a

difference in the membrane potential having mean η_{ext} and standard deviation Σ_{ext}. See Equation S27 and its derivation. Note that, in order to scale properly with the time step of the noise generator, the standard deviation depends on Δt_{ng}. The background input is the main external stimuli, which drives the spontaneous activity of the network. All the other excitatory stimuli depend on the parameters chosen for the background input according to a contrast factor A, so that the overall average current of the network during the injection of both background and the external stimuli in exam is $A\mu_{ext}$, with a standard deviation of σ_{ext}.

1.1 Changes of simulation time step and connectivity

This network model is designed in order to be similar as the one proposed in the original work of Mongillo et al. (2008), however some information regarding the network was missing. For instance, it is not known the simulation time step employed in the original work, in which both neuron and synaptic dynamics is integrated using the Euler scheme. In this work, the neuron model employed is integrated using the exact integration method of Rotter and Diesmann (1999). We decided to perform network simulations using a time step of $dt = 0.05$ ms, but we performed some tests in order to see whether a change in the value of the time step would have entailed significant difference in the network dynamics. In particular, we performed simulations with $dt = 0.1$ ms and $dt = 0.01$ ms. In the following Figure S1 it will be shown a similar simulation to the one proposed in Figure 2B of the manuscript, when the network is able to show an autonomous and synchronous spiking activity (i.e. the population spikes) using $dt = 0.1$ ms, $dt = 0.05$ ms (as in the rest of the manuscript) and $dt = 0.01$ ms.

As can be noticed, the behavior of the network using different simulation time steps is almost identical, with small differences regarding the time at which the population spikes occur. Also the histograms of the firing rate difference between the spontaneous activity and the delay period are totally comparable.

Regarding the connectivity, in NEST it is possible to have more than one connection between two neurons (called *multapses*) and self-connections (called *autapses*). We performed all the simulations enabling them (as it is the default option in the NEST simulator), however in the original model the possibility of having such connections was not specified. In the next figure it will be shown that, also disabling this option when building the model connectivity, the results are totally comparable.

1.2 Long-lasting simulations

The synchronous spiking activity and the asynchronous higher rate activity, shown in Figure 2B and 2C in the manuscript, can be stopped by diminishing the background input. To do so, we simply diminish the Gaussian white noise current in order to show the same values of current of Figure 2A, in which the memory reactivation is not spontaneous. Indeed, without this change in the mean value of the input current, the information is maintained in the selective population for an extremely long time. In this regard, we performed a long lasting simulation using the same parameters as in Figure 3B in the manuscript, which shows how two selective populations can keep two memories showing alternating population spikes.

As can be noticed, in Figure S3 are performed 3000 ms of pre-simulation and 20000 ms of simulation time, with no changes in the network dynamics after the loading of the memories.

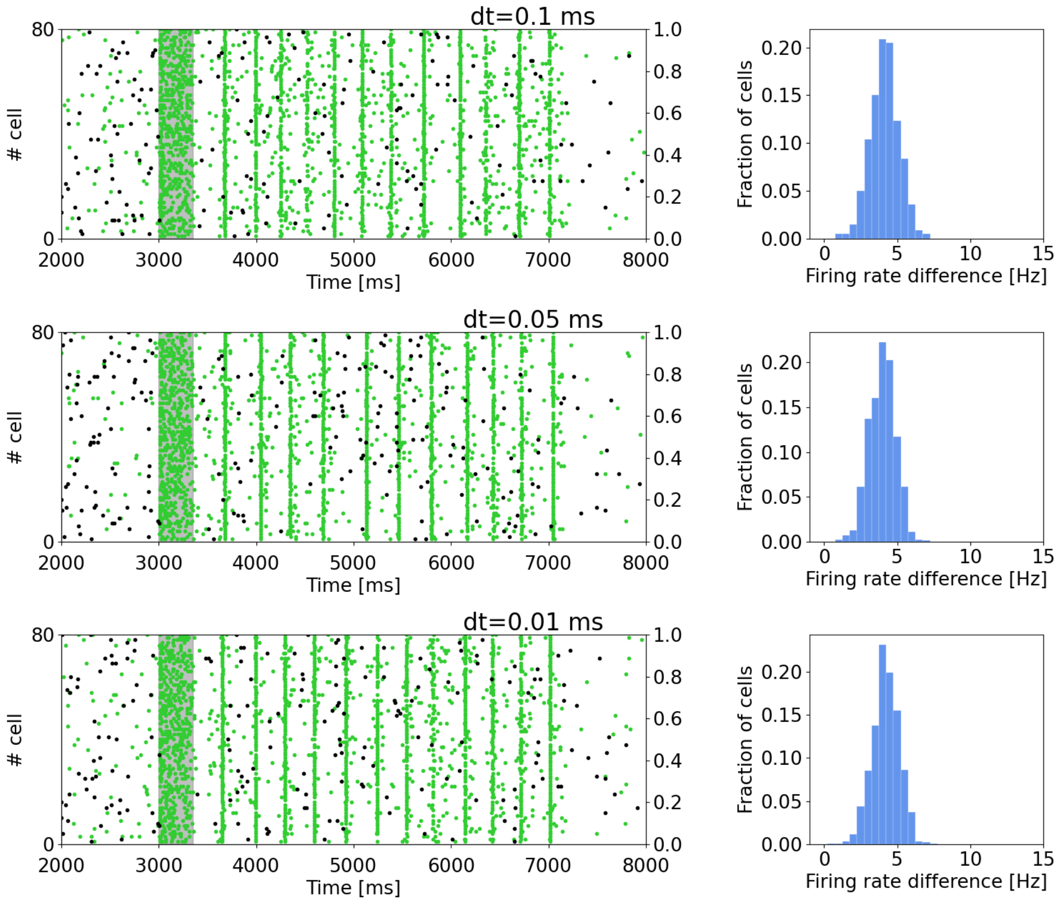

Figure S1: **(Left)** Raster plots of a subset of neurons of a targeted selective population (green) and a non-targeted one (black) for different values of simulation time step dt. **(Right)** Histograms representing the difference in firing rate between the delay period and the spontaneous state for the selective population targeted by the item loading signal. The external background input diminishes at 7.2 s.

2 STOCHASTIC INTEGRATION METHODS FOR NEURONAL DYNAMICS INTEGRATION: A RESULTS' COMPARISON

As reported in the manuscript, the LIF neuron model employed in the network is described by the ODE system:

$$\begin{aligned}\tau_m \frac{dV_j}{dt} &= -V_j + R_m(I_j^{exc} + I_j^{inh} + I_{ext,j}) \\ \tau_{exc} \frac{dI_j^{exc}}{dt} &= -I_j^{exc} + \sum_i \alpha J_{i,j}(t) \sum_s \delta(t - t_s^{(i)} - \hat{\delta}_{i,j}) \\ \tau_{inh} \frac{dI_j^{inh}}{dt} &= -I_j^{inh} + \sum_i \alpha J_{i,j} \sum_s \delta(t - t_s^{(i)} - \hat{\delta}_{i,j})\end{aligned} \tag{S1}$$

where τ_m is the membrane time constant, V_j is the neuron's membrane potential, R_m is the membrane resistance, I_j^{exc} and I_j^{inh} the excitatory and inhibitory synaptic current received as input from the connections within the other neurons of the network and $I_{ext,j}$ represents the external input to the network, modeled as a Gaussian white noise. The ODE system S1 is integrated following the exact integration

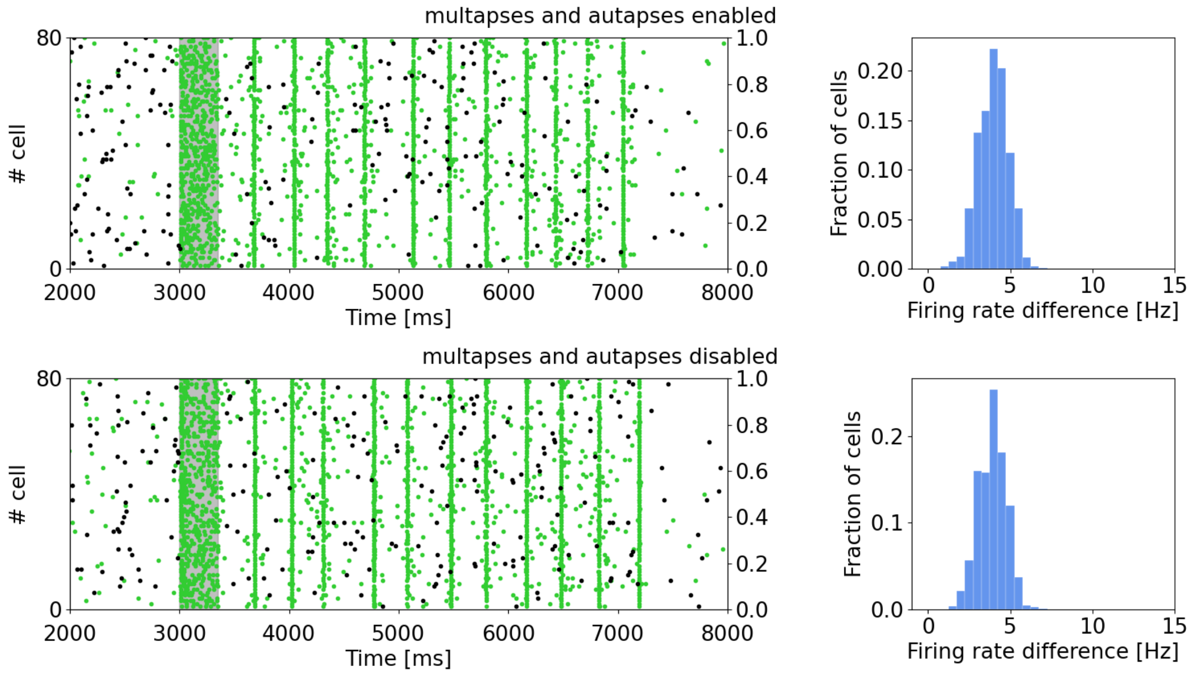

Figure S2: **(Left)** Raster plots of a subset of neurons of a targeted selective population (green) and a non-targeted one (black) enabling or disabling the possibility of having multiple connections between two neurons and self connections (called multapses and autapses respectively). **(Right)** Histograms representing the difference in firing rate between the delay period and the spontaneous state for the selective population targeted by the item loading signal. The external background input diminishes at $7.2\,\mathrm{s}$.

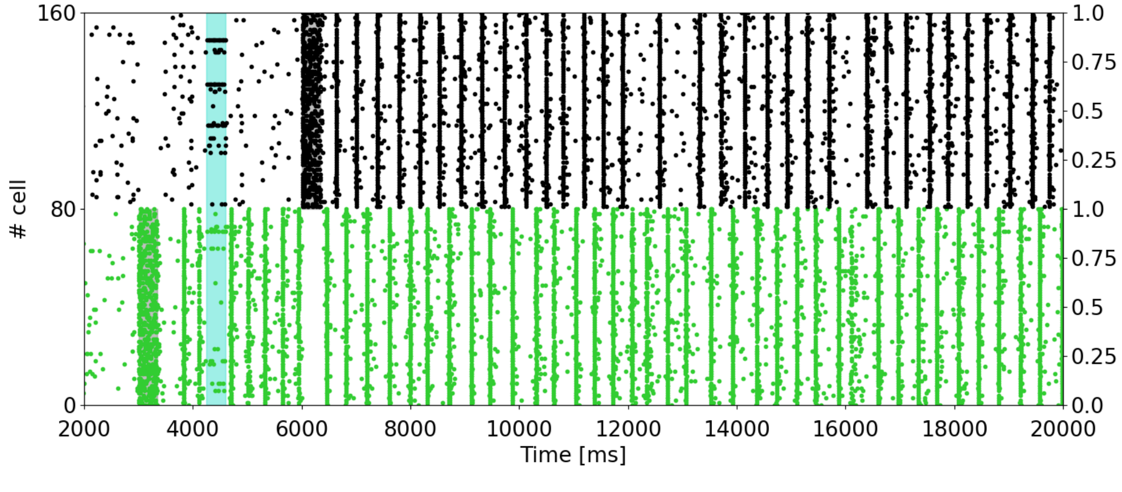

Figure S3: Raster plot of a subset of neurons of two targeted selective populations when two items are loaded into the network, with the network in the bi-stable state showing synchronous spiking activity. An additional noise (cyan shading), which targets the 15% of the excitatory neurons, is injected to test the network's robustness.

scheme of Rotter and Diesmann (1999), assuming that the external current $I_{ext,j}$ is piecewise constant over time intervals of width Δt_{ng}. An alternative approach can be developed by exploiting the theory of stochastic differential equations and the methods for their numerical solution.

Stochastic differential equations are generally defined as:

$$dX = a(t, X)dt + b(t, X)dW \tag{S2}$$

where dW represents the stochastic term and refers to the infinitesimal increment of the random walk of a Wiener process W, so that $dW = \sqrt{dt}G_n$, with G_n random number extracted from a Gaussian standard distribution. Terms $a(t, X)$ and $b(t, X)$ are respectively called drift coefficient and diffusion coefficient.

Indeed, the LIF model sub-threshold dynamics shown in Equation S1 can be described with a sum between a stochastic term (i.e. the Gaussian fluctuation of the external input) and a non-stochastic term. Expanding the term $I_{ext,j}$ and taking the finite difference of V_j over a small time step Δt, the first equation of the system S1 can be rewritten as:

$$\tau_m \Delta V_j = (-V_j + R_m I_j^{exc} + R_m I_j^{inh} + R_m \mu_{ext})\Delta t + R_m \sigma_{ext} G_n \Delta t \tag{S3}$$

where G_n is a random number extracted from a standard Gaussian distribution, whereas μ_{ext} and σ_{ext} are respectively mean and standard deviation of the external input signal. Defining $\sigma_{ext} = \kappa_{ext}/\sqrt{\Delta t}$, the last term in the left side of Equation S3 can be written as:

$$R_m \kappa_{ext} \Delta W_n \tag{S4}$$

where ΔW_k is the variation of a Wiener process

$$\Delta W_n = \sqrt{\Delta t} G_n \tag{S5}$$

Within each time step, equations for I_j^{exc} and I_j^{inh} can be solved analytically:

$$\begin{aligned} I_j^{exc}(t) &= I_j^{exc}(t_n) e^{-(t-t_n)/\tau_{exc}} \quad \text{for } t_n < t < t_{n+1} \\ I_j^{inh}(t) &= I_j^{inh}(t_n) e^{-(t-t_n)/\tau_{inh}} \quad \text{for } t_n < t < t_{n+1} \end{aligned} \tag{S6}$$

By substituting the solutions for $I_j^{exc}(t)$ and $I_j^{inh}(t)$, Equation S3 assumes the form of Equation S2 with $a(V_j, t) = (-V_j + R_m I_j^{exc} + R_m I_j^{inh} + R_m \mu_{ext})/\tau_m$ and $b = R_m \kappa_{ext}/\tau_m$.

Indeed, Equation S3 is a stochastic differential equation (SDE), which can be numerically integrated using specifically-designed numerical techniques. In this Section we present the comparison of the network behavior integrated following the exact scheme of Rotter and Diesmann (1999) (as in the manuscript) with respect to the behavior observed by using a specific SDE numerical integration technique, which will be presented in the next few lines.

Such a differential equation can be numerically integrated using the Euler-Maruyama method, which is an extension of the Euler method for SDE. According to this method, the solution for X in Equation S2 is defined as follows:

$$X(t_{n+1}) = X(t_n) + a(t_n, X(t_n))\Delta t + b(t_n, X(t_n))\Delta W_n \tag{S7}$$

where $\Delta W_n = \sqrt{\Delta t} G_n$, and the time interval $[0, T]$ in which the equation is integrated is divided into N equal intervals t_n, so that $t_{n+1} - t_n = \Delta t = T/N$. As can be noticed from Equation S7, this integration method leads to a systematic error, since drift and diffusion coefficient are considered constant over each

time interval $[t_n, t_{n+1}]$ with the values that they have at the beginning of the interval t_n. This error can be mitigated by reducing the time step, at the expense of the computational cost of the simulations. The Euler–Maruyama method has weak order of convergence 1, however it has strong order of convergence $1/2$ (Mackevičius, 2011).

A more precise class of methods has been derived through extensions of the Runge-Kutta method. In particular, Roberts (2012) proposes an implementation of the Runge-Kutta method for SDEs which has both weak and strong order of convergence 1, and does not produce the systematic error of the Euler–Maruyama method mentioned above. Given a SDE as the one of Equation S2, a time step Δt so that $t_{n+1} = t_n + \Delta t$ and the value $X(t_n) = X_n$, the value for the subsequent time step $X(t_{n+1}) = X_{n+1}$ is determined by:

$$X_{n+1} = X_n + \frac{1}{2}(K_1 + K_2) \tag{S8}$$

where

$$\begin{aligned} K_1 &= a(t_n, X_n)\Delta t + (\Delta W - S_n\sqrt{h})b(t_n, X_n) \\ K_2 &= a(t_{n+1}, X_n + K1)\Delta t + (\Delta W - S_n\sqrt{h})b(t_{n+1}, X_n + K1) \end{aligned} \tag{S9}$$

where $\Delta W_n = \sqrt{\Delta t}G_n$, with G_n random number extracted from a standard normal distribution and S_n is a coefficient whose value depends on the SDE integration scheme. In particular, the Itô integration scheme can be achieved by assigning S_n the values $+1$ and -1 with equal probability, while the Stratonovich integration scheme is obtained by setting $S_n = 0$ (Roberts, 2012). In this work we used the Stratonovich scheme, which is more appropriate for systems with external noise (van Kampen, 1981).

In Figure S4 the raster plot for the network simulation using SDE Runge-Kutta scheme is compared with respect to the simulation obtained using the exact integration method of Rotter and Diesmann (1999). In particular, the simulation is performed using the background current needed to show the synchronous spiking activity during the delay period (as the one shown in Figure 2B of the manuscript).

As can be noticed, the network behavior observed using the SDE Runge-Kutta integration method (top panel of Figure S4) is totally comparable to the behavior of the network obtained using the exact integration method of Rotter and Diesmann (1999) (bottom panel of Figure S4), which is the method used for all the simulations presented in the manuscript.

3 SHORT-TERM PLASTICITY MODEL

The STP model of Tsodyks et al. (1998, 2000) describes the behavior of the amount of available resources in the presynaptic terminal by using a system of three differential equations, together with the differential equation describing the behavior of the utilization factor u:

$$\begin{aligned} \frac{dx}{dt} &= \frac{z}{\tau_d} - u(t_s)x(t_s - \epsilon)\delta(t - t_s) \\ \frac{dy}{dt} &= -\frac{y}{\tau_{syn}} + u(t_s)x(t_s - \epsilon)\delta(t - t_s) \\ \frac{dz}{dt} &= \frac{y}{\tau_{syn}} - \frac{x}{\tau_d} \\ \frac{du}{dt} &= -\frac{u}{\tau_f} + U(1 - u)\delta(t - t_s) \end{aligned} \tag{S10}$$

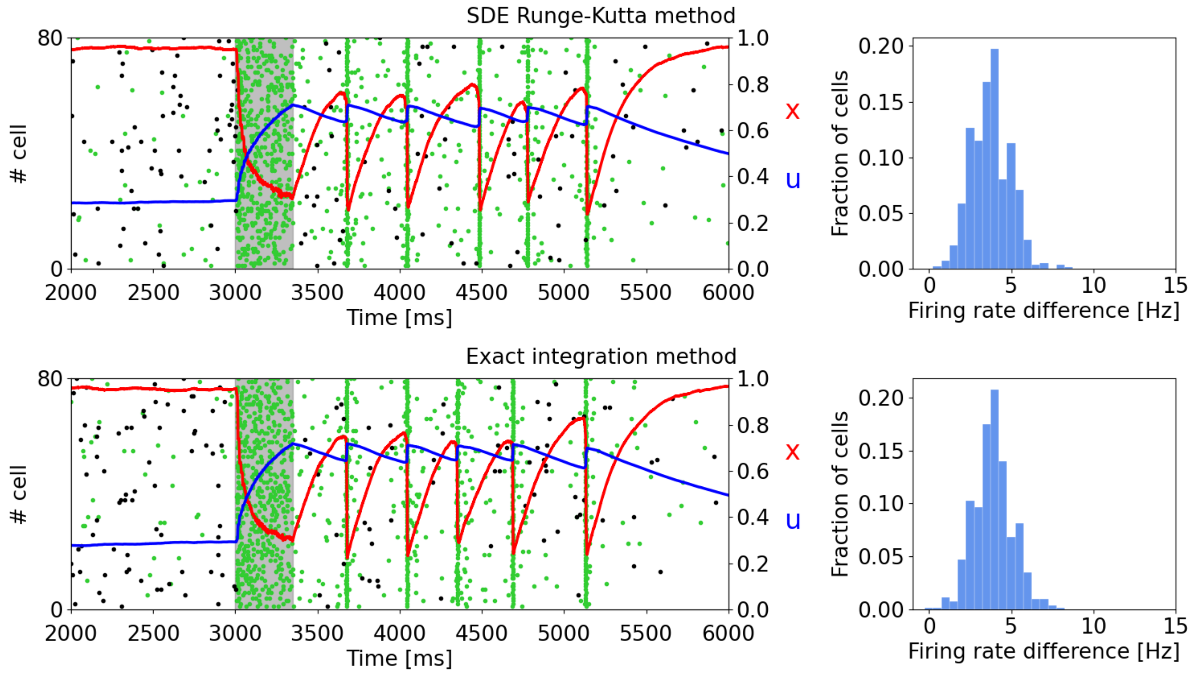

Figure S4: Simulations of the network using different numerical integration techniques. **(Left)** Raster plots of a subset of neurons of a targeted selective population (green) and a non-targeted one (black). **(Right)** Histograms representing the difference in firing rate between the delay period and the spontaneous state for the selective population targeted by the item loading signal.

where x, y and z are respectively the (normalized) amount of resources in the recovered, active state and inactive state. Here, the synaptic modulation is driven by the variable $y(t)$. In this equation and in the following, for simplicity, we neglect the indexed i, j indicating the presynaptic and the postsynaptic neuron. The model is already implemented in the NEST simulator under the name of `tsodyks_synapse` and it exactly solves equation S10. Is it possible to show that such a model can be simplified by adopting a system of two differential equations which describe the behavior of the synaptic resources (x) and the one of the utilization factor (u) (Tsodyks et al., 1998) so that

$$\begin{aligned} \frac{du}{dt} &= -\frac{u-U}{\tau_f} + U(1-u)\delta(t-t_s) \\ \frac{dx}{dt} &= \frac{1-x}{\tau_d} - ux\delta(t-t_s) \end{aligned} \tag{S11}$$

The model described in S11 is the same adopted in Barak and Tsodyks (2007) and Mongillo et al. (2008) (see equations 5 and 6 of the Supporting Material of Mongillo et al. (2008)). Further, such a model is included in NEST as well under the name of `tsodyks2_synapse`. As mentioned in the manuscript, the implementation is called when a spike has to be emitted, so it performs the temporal evolution from t_{s-1} and t_s and not at the end of every simulation step.

However, the model used in this work to describe STP dynamics is a modification of the latter NEST synapse model, which we named `tsodyks3_synapse`. Our modification involves only the order of the variables updates, in agreement with equation (3.1) of Tsodyks et al. (1998), which leads to a difference in synaptic modulation. With our modification, the model uses in the synaptic modulation the value of x

evolved from the time t_{s-1} to t_s (as in `tsodyks2_synapse`) and the value of u evolved in the same time interval summed with the factor $U(1-u)$. After the synaptic modulation, the variable x is thus updated by subtracting the quantity ux. The `tsodyks2_synapse` model, differently, modules the synaptic efficacy using the value of u without adding the factor $U(1-u)$, resulting in lower amplitudes in the postsynaptic currents.

To verify the reliability of `tsodyks3_synapse` model, we compare it with the NEST models `tsodyks_synapse` and `tsodyks2_synapse`. To do so, we simulate four LIF neurons with exponential postsynaptic currents (same model as the one used in the network): the first (presynaptic) neuron is injected with a constant current able to induce the emission of spikes during the first 500 ms and the last 500 ms of simulation. Between the two current injections a time interval of 1000 ms is simulated without injecting any current to observe the weight change after a short period of inactivity. The neuron is then connected to the other neurons using one of the three synaptic models with the same parameters. Then, the membrane potential of the three postsynaptic neurons is recorded to analyze the differences in the peak amplitude (i.e. of the modulated synaptic weight). The postsynaptic potentials are shown in Figure S5.

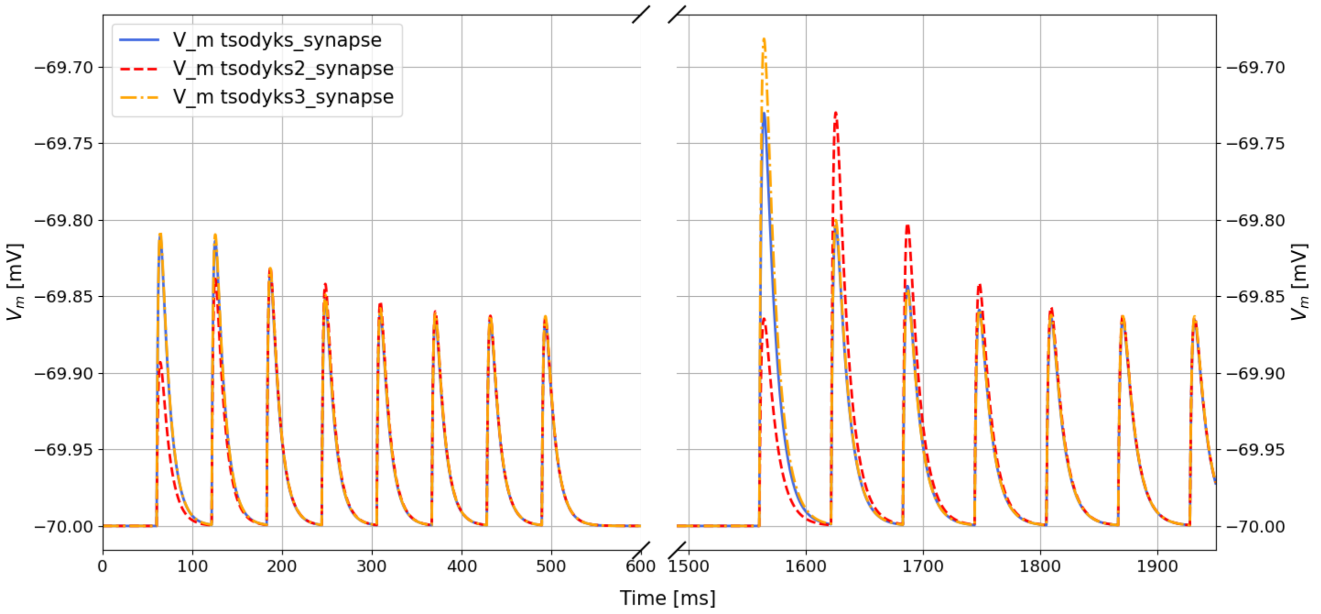

Figure S5: Excitatory postsynaptic potential of neurons connected to the same presynaptic neurons using different STP synapses. The blue line is obtained using the NEST synapse model `tsodyks_synapse`, the red dashed line is obtained using the `tsodyks2_synapse` and the orange line is obtained with `tsodyks3_synapse`. The time axis has been adjusted to show only the time intervals in which the presynaptic neuron is stimulated.

It is possible to notice that the difference in peak amplitude between the neurons is significant only for the first spikes, after which the models modulate the synaptic weights producing totally comparable postsynaptic potentials. The differences that arise between `tsodyks2_synapse` and its modification used in this work are only due to the difference in the order of update of the variable u and the synaptic weight. Moreover, the differences between such a model and the `tsodyks_synapse` are less significant and can be justified by the different behavior of u dynamics. In fact, the only relevant difference between these models can be observed after a relatively long-lasting time (in the order of τ_f) during which the STP

variable u can evolve and reach values near to the resting ones, which differs within the two models. In this regard, the first postsynaptic potential after the second of biological time during which the presynaptic neuron does not emit spikes shows the mentioned difference.

In summary, we decided to make this modification in order to be consistent with the order of variables update shown in equation (3.1) of Tsodyks et al. (1998). The modifications made of the `tsodyks2_synapse` model go in this direction, leading to a more comparable behavior with respect to the `tsodyks_synapse` model.

4 SYNAPTIC PARAMETERS AND WORKING MEMORY CAPACITY

As discussed in the article, the model simulated with the parameters used to reproduce Figure 2 and 3 (see Table S1 and Table S2) is able to keep at most three items at the same time. However, an increase in the value of τ_f enables the possibility of keeping more items. Here in Figure S6 is shown the raster plot of the selective populations of the model in a simulation in which τ_f has been increased so that the network is able to maintain all the five selective populations in the state of synchronous spikes emission.

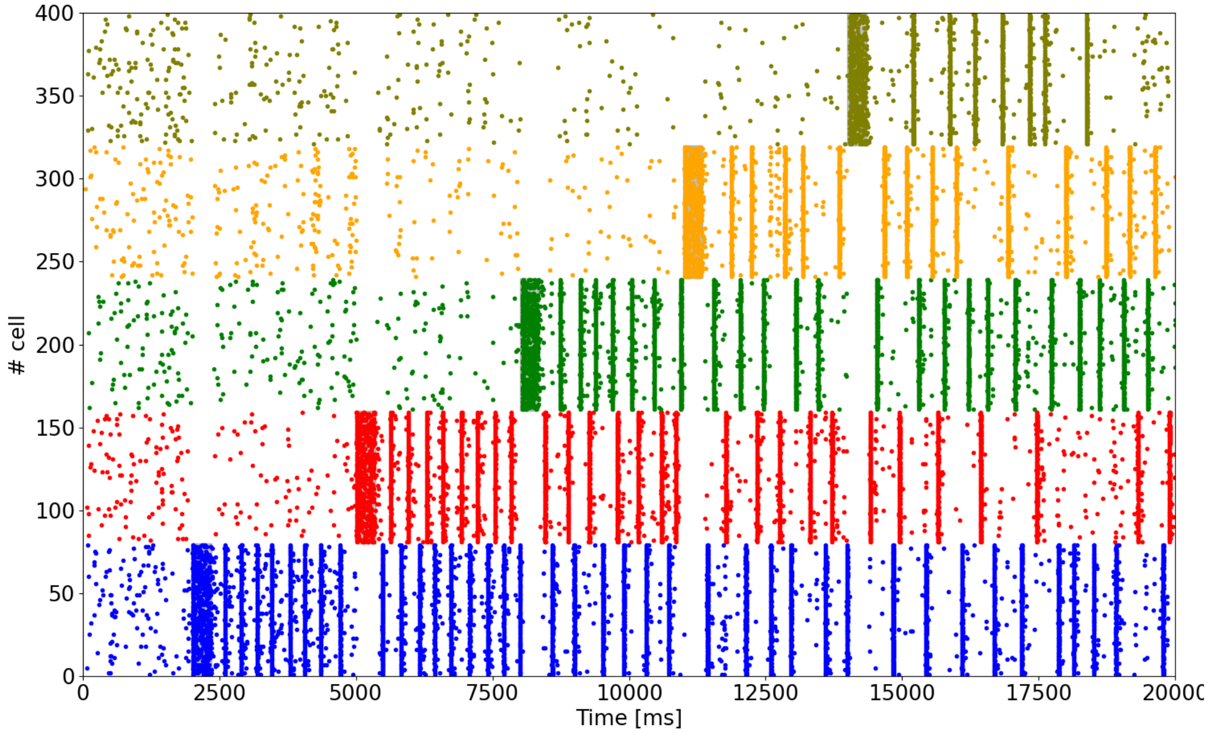

Figure S6: Raster plot of a subset of the five selective populations of the model. An item loading signal is injected every 3000 ms starting from 2000 ms to each selective population. The network, simulated using $\tau_f = 3000$ ms, is able to maintain all the five selective populations in the synchronous activity state.

The network model described in the article is composed of 10000 LIF neurons and has a total of five selective populations. Even if the number of selective populations is a model parameter, its change requires a network scaling in order to have a similar ratio between selective, non selective and inhibitory populations. In Mi et al. (2017) a network of 20000 LIF neurons and ten selective populations, designed as the one proposed in Mongillo et al. (2008), is described, so we decided to use similar parameters to perform

simulations with a larger network able to have an higher number of selective populations. To do so, we chose $f = 0.4$, $p = 10$, $N_E = 16000$, $N_I = 4000$, $J_b = 0.05$ (as in Mi et al. (2017)). A simulation with a single item loaded was performed to tune the external background input targeting the excitatory neurons in order to show synchronous spiking activity. We found that $\mu_{ext} = 26.5$ mV was able to show the desired behavior. We simulated the network using different values of τ_f to notice the change in the number of items which can be simultaneously maintained, and we observed a similar behavior discussed with the smaller network, so an higher value of τ_f is needed to keep a larger number of items in memory. In particular we observed that with $\tau_f = 4000$ ms, $\tau_D = 250$ ms seven memories can be loaded into the network at the same time. The raster plot of the selective populations in this configuration of the model is shown in Figure S7.

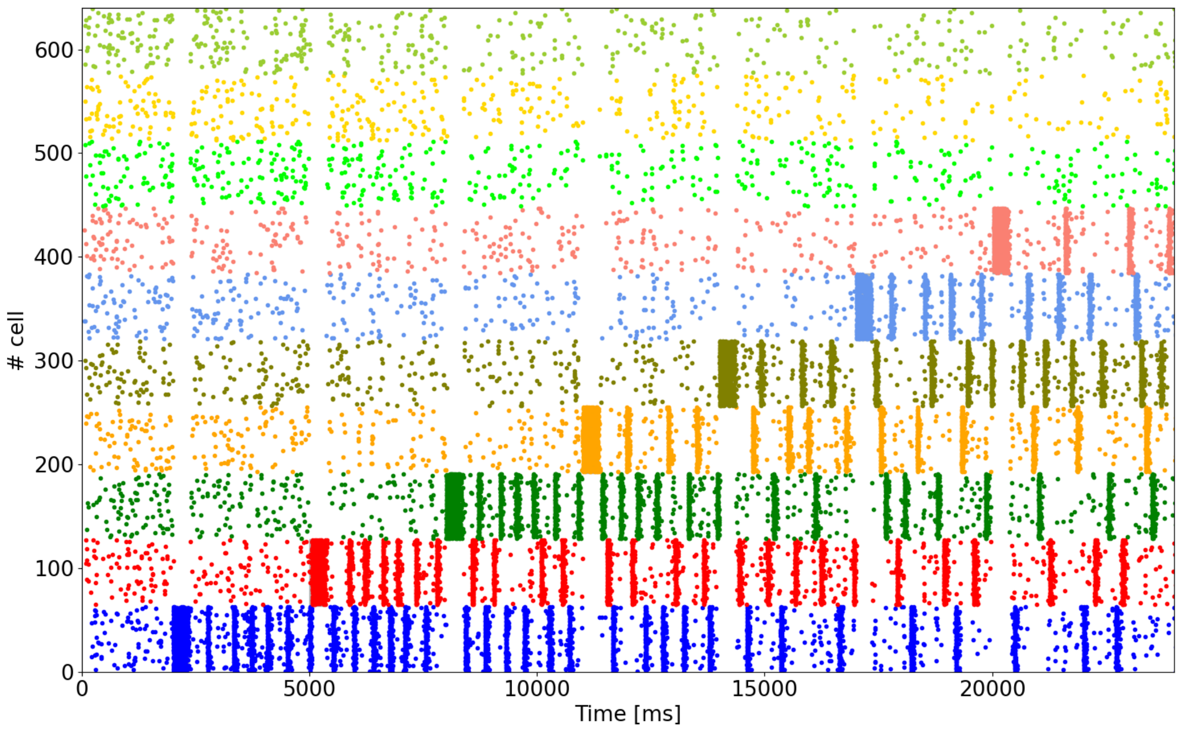

Figure S7: Raster plot of a subset of the ten selective populations of the model. An item loading signal is injected every 3000 ms for each of the first seven selective populations starting from 2000 ms. The network, simulated using $\tau_f = 4000$ ms, $\tau_d = 250$ ms, is able to maintain all the seven targeted selective populations in the synchronous activity state.

We tried to estimate the working memory capacity of such a network using the analytic expression shown in Mi et al. (2017). Using the parameters employed to obtain Figure S7 we have that the maximal period of the limit cycle of the network is $T_{max} \simeq 745$ ms, whereas the time between two successive population spikes after having loaded all the seven items is $t_s = 140(40)$ ms. Those values lead to an analytical estimation of working memory capacity between 4.1 and 7.5, with the average estimation being equal to 5.3, which is slightly smaller than the effective number of items kept in memory. However, it is still compatible with it considering the large uncertainty in this estimation.

5 RECORDING AND ANALYSIS OF STP VARIABLES

In NEST, STP variables can not be recorded using a proper recording device, therefore to permit the extraction of the values we perform the network simulation in steps, after each of which we record the values of u and x for the connections of the excitatory neurons. We decided to perform the simulation in steps of 10 ms to be able to have an adequate resolution to analyze the behavior of the variables and to grant a reasonable computing time.

Moreover, since the STP variables are updated only when a spike has to be emitted, the temporal evolution of the variables has to be computed separately. We compute the variable's temporal evolution using the analytic solution of the differential equations of u and x (see equation S11) for the steps in which no spikes are fired.

After computing the evolution of x and u for every neuron we average the variables over the selective population to have an estimation of the synaptic behavior of the neuron's population.

We adopted this method since this way we record the parameter values used by the spiking network instead of computing the entire behavior of the STP variables separately using the neuron's spike times. However, the latter approach surely would grant a substantial advantage in the network simulation time, since the recording of STP variables leads to a relevant slow-down of the simulation.

6 FINDING THE CURRENT NEEDED TO ELICIT A PSP

In Table S1 are illustrated the synaptic parameters used in this spiking network model. It should be noted that the synaptic efficacies are expressed in mV and not in pA, that is the unit needed by the NEST simulator for current-based neuron models. To obtain the analytic expression for the value of current needed to elicit a given postsynaptic potential, the equation describing the sub-threshold dynamics of the LIF neuron model and the behavior of the postsynaptic current are needed. In particular we have

$$\tau_m \frac{dV}{dt} = -V + RI_0 \tag{S12}$$

where $R = \tau_m/C_m$ and $I_0 \propto e^{-t/\tau_s}$. τ_m and τ_s represents the membrane time constant and the synaptic current time constant respectively. The general solution of Equation S12, considering the exponential behavior of I_0 is

$$V(t) = V_0 e^{-t/\tau_m} + \frac{\tau_m}{C_m}\frac{\tau_s}{\tau_s - \tau_m} I_0 e^{-t/\tau_s} \tag{S13}$$

and considering $V(0) = 0$

$$V(t) = -\frac{\tau_m}{C_m}\frac{\tau_s}{\tau_s - \tau_m} I_0 e^{-t/\tau_m} + \frac{\tau_m}{C_m}\frac{\tau_s}{\tau_s - \tau_m} I_0 e^{-t/\tau_s} \tag{S14}$$

The Equation S14 represents the postsynaptic response to a current stimulus of amplitude I_0 reached at $t = 0$. Imposing the derivative of Equation S14 to zero enables us to find the time t_{max} at which the membrane reaches the maximum depolarization because of the stimulus applied. So we have

$$t_{\text{max}} = \ln\left[\left(\frac{\tau_m}{\tau_s}\right)^{\tau_m\tau_s/(\tau_m-\tau_s)}\right] \tag{S15}$$

and

$$V(t_{\text{max}}) = \frac{\tau_m\tau_s I_0}{C_m(\tau_s - \tau_m)}\left[\left(\frac{\tau_m}{\tau_s}\right)^{-\tau_m/(\tau_m-\tau_s)} - \left(\frac{\tau_m}{\tau_s}\right)^{-\tau_s/(\tau_m-\tau_s)}\right] \tag{S16}$$

The equation above represents the amplitude of the postsynaptic potential elicited by a postsynaptic current of amplitude I_0. Thus, the value of I_0 needed to obtain a postsynaptic potential amplitude of $V(t_{\text{max}})$ is

$$\alpha = \frac{I_0}{V(t_{\text{max}})} = \left\{ \frac{\tau_m \tau_s}{C_m(\tau_s - \tau_m)} \left[\left(\frac{\tau_m}{\tau_s}\right)^{-\tau_m/(\tau_m - \tau_s)} - \left(\frac{\tau_m}{\tau_s}\right)^{-\tau_s/(\tau_m - \tau_s)} \right] \right\}^{-1} \tag{S17}$$

which is the analytic expression used in this model to get the value of the synaptic efficacy needed to elicit the potentials illustrated in Table S1. In fact, the term α represents the variation of input current needed to have a unit of variation of the postsynaptic potential.

7 FINDING THE EXTERNAL CURRENT NEEDED TO ELICIT A GIVEN AVERAGE AND STANDARD DEVIATION OF THE MEMBRANE POTENTIAL

In Table S2 are illustrated the synaptic parameters used in this spiking network model to build the external stimuli. It should be noted that the external stimulus, modeled as a Gaussian white noise current, shows mean η_{ext} and standard deviation Σ_{ext} expressed in mV and not in pA. In this section it will be derived the expression of the average and standard deviation a current signal should have to elicit an average membrane potential η with standard deviation Σ. This derivation can also be found in the NEST Documentation together with tests and examples using the NEST *noise_generator*, which is used in this model to inject the Gaussian white noise current to the neurons of the network.

The sub-threshold dynamics of a LIF neuron model is given by Equation S12. As explained in the manuscript, the Gaussian white noise current is a piecewise constant current which changes at fixed intervals Δt_{ng}. The current has average μ and standard deviation σ, in pA units. The autocorrelation of the current is given by

$$\langle (I_k - \mu)(I_m - \mu) \rangle = \sigma^2 \delta_{km}$$

where δ_{km} is a Dirac delta. Defining $V_n = V(t_n)$ the membrane potential of a neuron at the time t_n and I_n the value of the constant current in the time step $\Delta t_{ng} = t_{n+1} - t_n$, the membrane potential at time t_{n+1} is:

$$V_{n+1} = V_n e^{-\Delta t_{ng}/\tau_m} + \frac{I_n \tau_m}{C_m}(1 - e^{-\Delta t_{ng}/\tau_m}) \tag{S18}$$

Setting $V_0 = 0$ mV and applying the previous equation recursively backwards the equation becomes:

$$V_{n+1} = \left[V_{n-1} e^{-\Delta t_{ng}/\tau_m} + \frac{I_{n-1}\tau_m}{C_m}(1 - e^{-\Delta t_{ng}/\tau_m})\right] e^{-\Delta t_{ng}/\tau_m} + \frac{I_n \tau_m}{C_m}(1 - e^{-\Delta t_{ng}/\tau_m}) \tag{S19}$$

and thus:

$$V_{n+1} = (1 - e^{-\Delta t_{ng}/\tau_m}) \frac{\tau_m}{C_m} \sum_{i=0}^{n} I_n e^{n\Delta t_{ng}/\tau_m} \tag{S20}$$

Using Equation S20 it is possible to find the average η and the standard deviation Σ of the membrane potential of a LIF neuron given a current with mean μ and standard deviation σ. This way, by inverting the expressions, it is possible to find the current input parameters needed.

Given that $\langle I_n \rangle = \mu$ and $\sum_{i=0}^{n} e^{-i\alpha} = (1 - e^{-(n+1)\alpha})/(1 - e^{-\alpha})$, the mean of the membrane potential at the time t_{n+1} is:

$$\begin{aligned}\langle V_{n+1} \rangle &= (1 - e^{-\Delta t_{ng}/\tau_m}) \frac{\tau_m}{C_m} \sum_{i=0}^{n} \langle I_i \rangle e^{i\Delta t_{ng}/\tau_m} = \\ &= (1 - e^{-\Delta t_{ng}/\tau_m}) \mu \frac{\tau_m}{C_m} \frac{1 - e^{-(n+1)\Delta t_{ng}/\tau_m}}{1 - e^{-\Delta t_{ng}/\tau_m}} = \\ &= \mu \frac{\tau_m}{C_m} (1 - e^{-(n+1)\Delta t_{ng}/\tau_m}) = \mu \frac{\tau_m}{C_m} (1 - e^{-t_{n+1}/\tau_m})\end{aligned} \tag{S21}$$

For $t \to \infty$ the average membrane potential is:

$$\lim_{t \to \infty} \langle V(t) \rangle = \eta = \mu \tau_m / C_m \tag{S22}$$

thus, given η (as in Table S2), the mean current is $\mu = \eta C_m / \tau_m$.

To find the expression for the variance of the membrane potential we need to compute the second moment $\langle V_{n+1}^2 \rangle$:

$$\begin{aligned}\langle V_{n+1}^2 \rangle =& \frac{\tau_m^2}{C_m^2} (1 - e^{-\Delta t_{ng}/\tau_m})^2 \Big\langle \Big(\sum_{i=0}^{n} I_i e^{-i\Delta t_{ng}/\tau_m} \Big)^2 \Big\rangle = \\ =& \beta \Big\langle \Big(\sum_{i=0}^{n} I_i \gamma^i \Big)^2 \Big\rangle = \\ =& \beta \sum_{i=0}^{n} \sum_{j=0}^{n} \langle I_i I_j \rangle \gamma^{i+j} = \beta \sum_{i=0}^{n} \sum_{j=0}^{n} (\mu^2 + \sigma^2 \delta_{ij}) \gamma^{i+j} = \\ =& \beta \mu^2 \Big(\sum_{i=0}^{n} \gamma^i \Big)^2 + \beta \sigma^2 \sum_{i=0}^{n} \gamma^{2i} = \\ =& \langle V_{n+1} \rangle^2 + \beta \sigma^2 \sum_{i=0}^{n} \gamma^{2i}\end{aligned} \tag{S23}$$

where $\beta = \frac{\tau_m^2}{C_m^2} (1 - e^{-\Delta t_{ng}/\tau_m})^2$ and $\gamma = e^{-\Delta t_{ng}/\tau_m}$.

Thus the variance of the membrane potential is:

$$\begin{aligned}\langle \Delta V_{n+1}^2 \rangle =& \beta \sigma^2 \sum_{i=0}^{n} \gamma^{2i} = \frac{\tau_m^2}{C_m^2} \sigma^2 (1 - \gamma)^2 \sum_{i=0}^{n} \gamma^{2i} = \\ =& \frac{\tau_m^2}{C_m^2} \sigma^2 (1 - \gamma)^2 \frac{\gamma^{2(n+1)} - 1}{\gamma^2 - 1} = \\ =& \frac{\tau_m^2}{C_m^2} \sigma^2 (1 - \gamma^{2(n+1)}) \frac{1 - \gamma}{1 + \gamma} = \\ =& \frac{\tau_m^2}{C_m^2} \sigma^2 \frac{1 - e^{-\Delta t_{ng}/\tau_m}}{1 + e^{-\Delta t_{ng}/\tau_m}} (1 - e^{-2t_{n+1}/\tau_m})\end{aligned} \tag{S24}$$

For $t \to \infty$ the variance of the membrane potential becomes:

$$\lim_{t\to\infty} \langle (\Delta V(t))^2 \rangle = \Sigma^2 = \frac{\tau_m^2}{C_m^2}\sigma^2 \frac{1 - e^{-\Delta t_{ng}/\tau_m}}{1 + e^{-\Delta t_{ng}/\tau_m}} \tag{S25}$$

and for $\Delta t_{ng} \ll \tau_m$ the variance is:

$$\Sigma^2 \approx \frac{\tau_m}{2C_m^2}\sigma^2 \Delta t_{ng} \tag{S26}$$

thus, given the standard deviation Σ (as in Table S2), the standard deviation of the input current signal is $\sigma = \sqrt{2/(\tau_m \Delta t_{ng})} C_m \Sigma$.

In conclusion, given the mean η and the standard deviation Σ of the membrane potential of a neuron injected with a piecewise constant current of mean μ and standard deviation Σ changing at fixed intervals of length Δt_{ng}, the input current parameters can be computed as follows:

$$\begin{aligned} \eta &= \mu \frac{C_m}{\tau_m} \\ \Sigma &= \sqrt{\frac{2}{\tau_m \Delta t_{ng}}} C_m \sigma \end{aligned} \tag{S27}$$

REFERENCES

Barak, O. and Tsodyks, M. (2007). Persistent activity in neural networks with dynamic synapses. *PLOS Computational Biology* 3, 1–1. doi:10.1371/journal.pcbi.0030035

Mackevičius, V. (2011). *Introduction to Stochastic Analysis* (John Wiley & Sons, Ltd). doi:https://doi.org/10.1002/9781118603338

Mi, Y., Katkov, M., and Tsodyks, M. (2017). Synaptic correlates of working memory capacity. *Neuron* 93, 323–330. doi:https://doi.org/10.1016/j.neuron.2016.12.004

Mongillo, G., Barak, O., and Tsodyks, M. (2008). Synaptic theory of working memory. *Science* 319, 1543–1546. doi:10.1126/science.1150769

Roberts, A. J. (2012). Modify the improved Euler scheme to integrate stochastic differential equations doi:10.48550/ARXIV.1210.0933

Rotter, S. and Diesmann, M. (1999). Exact digital simulation of time-invariant linear systems with applications to neuronal modeling. *Biological Cybernetics* 81, 381–402. doi:10.1007/s004220050570

Tsodyks, M., Pawelzik, K., and Markram, H. (1998). Neural Networks with Dynamic Synapses. *Neural Computation* 10, 821–835. doi:10.1162/089976698300017502

Tsodyks, M., Uziel, A., and Markram, H. (2000). Synchrony generation in recurrent networks with frequency-dependent synapses. *Journal of Neuroscience* 20, RC50–RC50. doi:10.1523/JNEUROSCI.20-01-j0003.2000

van Kampen, N. G. (1981). Itô versus Stratonovich. *Journal of Statistical Physics* 24, 175–187. doi:10.1007/bf01007642
